# DNA methylation in canine brains is related to domestication and dog-breed formation

**DOI:** 10.1371/journal.pone.0240787

**Published:** 2020-10-29

**Authors:** Ann-Sofie Sundman, Fábio Pértille, Luiz Lehmann Coutinho, Elena Jazin, Carlos Guerrero-Bosagna, Per Jensen

**Affiliations:** 1 AVIAN Behaviour Genomics and Physiology Group, IFM Biology, Linköping University, Linköping, Sweden; 2 Animal Biotechnology Laboratory, Animal Science and Pastures Department, University of São Paulo (USP)/ Luiz de Queiroz College of Agriculture (ESALQ), Piracicaba, São Paulo, Brazil; 3 Department of Organismal Biology, EBC, Uppsala University, Uppsala, Sweden; Institute of Animal Science, CZECH REPUBLIC

## Abstract

Epigenetic factors such as DNA methylation act as mediators in the interaction between genome and environment. Variation in the epigenome can both affect phenotype and be inherited, and epigenetics has been suggested to be an important factor in the evolutionary process. During domestication, dogs have evolved an unprecedented between-breed variation in morphology and behavior in an evolutionary short period. In the present study, we explore DNA methylation differences in brain, the most relevant tissue with respect to behavior, between wolf and dog breeds. We optimized a combined method of genotype-by-sequencing (GBS) and methylated DNA immunoprecipitation (MeDIP) for its application in canines. Genomic DNA from the frontal cortex of 38 dogs of 8 breeds and three wolves was used. GBS and GBS-MeDIP libraries were prepared and sequenced on Illuma HiSeq2500 platform. The reduced sample represented 1.18 ± 0.4% of the total dog genome (2,4 billion BP), while the GBS-MeDIP covered 11,250,788 ± 4,042,106 unique base pairs. We find substantial DNA methylation differences between wolf and dog and between the dog breeds. The methylation profiles of the different groups imply that epigenetic factors may have been important in the speciation from dog to wolf, but also in the divergence of different dog breeds. Specifically, we highlight methylation differences in genes related to behavior and morphology. We hypothesize that these differences are involved in the phenotypic variation found among dogs, whereas future studies will have to find the specific mechanisms. Our results not only add an intriguing new dimension to dog breeding but are also useful to further understanding of epigenetic involvement.

## Introduction

During animal domestication, wild species are adapted to a life in human proximity. Through correlated selection responses to tameness selection, differences in phenotype can be acquired in only a few generations [1–3]. Even though behavior is profoundly modified during domestication, differences in DNA sequence between domesticates and their wild ancestor species are relatively few, while differences in brain gene expression are all the larger [4]. For example, SNP’s in dogs differing from wolves represent less than 0.1% of the entire genome [5, 6]. Although there are large technical challenges in identifying potential trait-related genetical variation, e.g. structural variants, the relatively small genetic variation between domesticates and their ancestors opens the possibility that epigenetic factors may play an important role in shaping phenotypic differences emerging in a short evolutionary time.

Epigenetic factors, e.g. DNA methylation, affect gene expression without altering the DNA sequence [7], and as this process is dynamic it can allow for plastic and adaptive responses to changes or challenges in the environment. Variation in DNA-methylation has been shown to affect various phenotypes, [e.g. HPA axis reactivity: [8], coat color: [9], social behavior: [10]]. Furthermore, it is to some extent heritable [11–13], and may promote genetic mutations [14]. Additionally, epigenetically controlled phenotypes can respond to selection [15]. It is thus not surprising that epigenetics has been suggested to be an important factor in evolutionary processes, including domestication [7, 16, 17]. Previously, DNA methylation patterns between domesticated chickens and their ancestor, the Red Junglefowl, have been found to differ substantially [18]. Also, recent research suggests that CpG-related mutations, specifically those linked to SNPs, have been important for speciation in chickens [19]. Specifically, CpGs associated with human-specific functional epigenetic changes (so-called ‘CpG Beacons’) in the prefrontal cortex of humans have been shown to have played an important role in evolution [20].

Wolves (*Canis lupus*) and domestic dogs (*Canis familiaris*) diverged more than 15,000 years ago [21]. From the proto-dog, breed types of different appearances and for different functions emerged and the breed diversification has intensified over the last 200 years [22]. Today, there are more than 400 breeds displaying an impressive variation in morphological and behavioral traits. Due to the modern breeding practice, breeds are mainly mated within the breed, and are thus isolated populations [22, 23]. Therefore, canines have been shown to be suitable study object for studying the role of genetic factors in selection processes and phenotypic differences, and may be suitable for studies of epigenetic factors as well.

Dog breeds have been selected not only for appearance, but above all for different behavior. The behavioral variation found today is staggering, with breeds specialized on, for example, herding, retrieving, guarding and hunting [24]. The dog differs in gene-expression patterns from their wild ancestor, the wolf [25, 26], although no substantial DNA-sequence differences can be found [27]. In concordance with studies in chickens, DNA methylation differences between wolf and dog have been identified when comparing blood and buccal samples [28] and these differences are often found in promotors for behavior-related genes [29]. However, DNA-methylation is highly tissue specific [30], and from a behavioral perspective, the brain should therefore be the focus organ to study. Here, we therefore focus for the first time on a broad analysis of epigenetic variation in the canine brain.

Although variation in only a few genes can have a large phenotypic impact on dog diversity [e.g. 31], it remains plausible that also epigenetics is involved in breed diversification. In line with this, Banlaki, Cimarelli [29] found breed-specific methylation patterns for behavior-related genes and a study on brain DNA methylation suggests breed differences in the monoamine oxidase A gene [10]. More comprehensive studies comparing brain DNA methylation between different dog breeds are, however, lacking.

Here, we have used brain samples from wolves and dogs to explore DNA methylation differences both across and within. Previous studies have found sex-specific DNA methylation profiles in several species [32, 33], therefore we explore this also in the canines. We show that there are substantial differences in brain DNA methylation profiles of wolf and dog, but also breed-specific patterns. This implies the importance of epigenetic differences for behavioural variation related to dog domestication and breed formation specifically, and for evolution and speciation in general.

## Material and methods

### Ethical statement

Canine brains were donated by their owners (dogs) and by the zoo (wolves), and all samples were collected in connection with veterinary motivated procedures. No ethical license was therefore required.

### Subjects and sampling

Brain tissue from 41 canine individuals was used in this study; three female wolves (*Canis lupus*) and 38 dogs (*Canis familiaris*) of eight different breeds, which represents the samples available at the time. The breeds were beagle (males: 3, females: 3), boxer (M: 1, F: 1), German shepherd dog (M: 3, F: 1), Great Dane (M: 5, F: 1), Labrador retriever (M: 2, F: 1), Pitbull terrier (M: 3, F: 4), Rottweiler (M: 2, F: 2) and walker hound (M: 3, F: 3). Dog samples were donated by owners after the dogs had been euthanized by veterinarians after decision by the owner. Samples were collected by Dog Genetics Project, Swedish University of Agricultural Sciences and Uppsala University, in connection to a previous study [25]. The ages of the dogs varied from one to ten years, with the exception of one rottweiler (3 months), and three German shepherds (6, 7 and 8 months). None of the dogs had been euthanized due to any brain disease, and the most common reasons were tumors outside of the nervous system and cardiovascular diseases. None of the dogs had metastases in the brains or circulating tumor emboli according the autopsy protocols. The brains were stored in a -80 freezer at Uppsala University until our sample dissection.

Three wolf brain samples were donated from Borås Zoo in connection with routine euthanasia of two 2 years old and one 9 year old surplus animals.

For the present study, a piece of tissue was dissected from the medial prefrontal cortex of the left cerebral hemisphere. This represents a part of the brain generally believed to be involved in cognitive aspects of behaviour. The brain tissue was kept frozen either on dry ice, in liquid nitrogen or in a -80 freezer until DNA extraction.

### DNA isolation

For DNA extraction, we used Qiagen All-Prep RNA/DNA kit and followed the instruction from the manufacturer. DNA quality was measured using ThermoFischer Scientific NanoDrop® ND-2000c and concentration was quantified using a fluorometer (Qubit® Fluorometric Quantitation).

### GBS and MeDIP

DNA from the prefrontal cortex was used for the genomic and epigenomic analyses. Because pyramidal cells are the most common neurons in the cerebral cortex, being its building block [34], it is expected that most of the epigenetic signal obtained belongs to these cells. To identify differences in methylation patterns, we have used a novel approach that combines genotyping by sequencing (GBS) [35] and methylated DNA immunoprecipitation (MeDIP) [36]. This method has previously been used in chickens (*Gallus gallus*) [37] and is here further optimized for application in canines based on *in silico* and *in vitro* digestion tests. Reducing the genome and pooling barcoded individual samples makes this a cost-efficient genotyping and epigenotyping method that allows comparisons of methylation profiles. Importantly, the GBS reduced representation method cleaves the genome at the same recognition sites in all individuals, which results in a non-random small fraction that constitutes a broadly representative genome sample. This sample can then be used for phylogenetic comparisons and also as input to represent the genetic background of each individual in the methylation analysis. The restriction sites used by the restriction enzyme *PstI* are unrelated to CpGs and, consequently, the positions are unbiased towards CpG islands.

To give an overview of the GBS method, the DNA is cleaved using *PstI* (Thermo Scientific) and enriched for fragments sized 200–500 basepairs (bp) long, suitable for Illumina sequencing. A DNA barcode, unique for each individual sample, together with a common adapter for Illumina sequencing barcoding system, is ligated to the fragments (Poland and Rife, 2012). The output of the barcoding and pooling is used as input for MeDIP. Two libraries are then prepared, one GBS library and one GBS-MeDIP library, and both libraries are sequenced. In our samples, sequencing was paired-end sequenced (read length: 125 bp) on the Illumina HiSeq2500 platform and performed at SNP&SEQ Technology Platform, SciLifeLab, Uppsala, Sweden. A more detailed description of the procedure can be found in S1 Table.

### Bioinformatic analyses

We used CASAVA (Illumina) for the initial processing of the samples and converted ".bcl" (base call files) to ".fastq" extensions. These are compatible with programs used for alignment reading. The quality of the reads was checked using FastQC v.0.11.3 34 and we performed quality trimming in short read sequences. Quality-trimmed reads were aligned against the canine reference genome (CanFam3, NCBI) using Bowtie2 tool v.2-2.2.9 [38] with default parameters for very-sensitive-local alignment. Coverage depth was determined by using Samtools v.1.3.1 [39].

TASSEL-GBS Discovery Pipeline [40] was used to process the GBS data. For SNP calling, default filtering parameters were used, except for 5% for minimum minor allele frequency (mnMAF), 70% of minimum taxon coverage (mnTCov), and 70% for minimum site coverage (mnScov). With Tassel we checked for allele changes between wolves and dogs and among dog breeds by comparing the number of fixed SNPs within each group. Tassel was additionally used to create a cladogram. It was generated by Neighbor Joining distance matrix and was plotted using Archaeopteryx tree.

For epigenetics analysis, reads from fastq files were demultiplexed using Stacks v.1.46 [41]. Uncalled and low-quality score bases were eliminated using process radtags function from Stacks. MEDIPS R-package from Bioconductor [42] was used for basic data processing, quality controls, normalization, and identification of differentially methylated regions (DMRs). We followed the same specific parameters from MEDIP package as described previously in Pértille, Brantsæter [37]. The genome was divided into adjacent windows of pre-defined length size of 100 bp and differential methylation analysis used a weighted trimmed mean of the log expression ratios (trimmed mean of M values) [43].

We compared DNA methylation of the adjacent 100 bp windows between several groups: 1) wolves were compared to all female dogs, 2) wolves were compared to females of each breed, 3) female dogs were compared to male dogs, and 4) breeds were compared to breeds, including both male and female individuals. The reason for only comparing wolves to female dogs was that our samples only included female wolves, and we wanted to avoid finding sex-specific effects instead of species effects. DMRs with p<0.0005 were considered significant. Manhattan plots were created using the R package qqman [44].

The DMRs were annotated against the dog reference genome (CanFam3, NCBI) using the Variant Effect Predictor (VEP) tool [45]. The annotations were analyzed through different online bioinformatic tools. Reactome pathway browser [[46]; reactome.org] was used to find overrepresented pathways related to genes in the wolf-dog comparison. An overrepresentation enrichment gene ontology analysis was performed using WEB-based GEne SeT AnaLysis Toolkit (WebGestalt) [[47]; webgestalt.org]. For both analyses, DMRs p<0.05 were used and the dog genome was used as background. Gene information was retrieved using PANTHER [[48]; pantherdb.org] and for high level gene ontology categories for groups of genes, we used ShinyGO v0.50 [[49]; bioinformatics.sdstate.edu/go/].

## Results

### Sequencing results

On average, the GBS resulted in a coverage of 28,226,049 ± 9,895,466 (SD) unique base pairs (breadth) which represents 1.18 ± 0.4% of the total dog genome (2,4 billion BP). The GBS-MeDIP covered 11,250,788 ± 4,042,106 unique base pairs which represents 0.47 ± 0.17% of the total genome. Table 1 presents average coverage for each dog breed and the wolf for both GBS and GBS-MeDIP.

**Table 1:** Average coverage for GBS and GBS-MeDIP for all samples.

	GBS				GBS-MeDIP			
	Depth	BP seq	Breadth	%	Depth	BP seq	Breadth	%
**TOTAL**	**33.2**	**1,049,816,037**	**28,226,048**	**1.18**	**44.8**	**553,517,692**	**11,250,788**	**0.47**
Beagle	31.6	998,800,150	29,853,945	1.25	43.9	527,827,555	11,819,563	0.49
Boxer	29.6	614,887,698	20,763,844	0.87	33.2	243,807,962	7,359,138	0.31
German sh.	30.5	673,323,948	20,019,369	0.84	40.8	295,928,883	7,123,554	0.30
Great Dane	40.1	996,421,318	24,141,234	1.01	45.2	449,581,812	9,504,467	0.40
Labrador	22.5	494,016,535	21,733,556	0.91	24.8	230,979,636	9,477,432	0.40
Pitbull	38.5	1,593,561,439	34,791,126	1.45	53.4	923,696,054	15,236,931	0.64
Rottweiler	31.9	1,068,933,565	31,149,168	1.30	52.0	576,496,251	10,710,978	0.45
Walker	34.3	1,325,354,578	33,115,536	1.38	50.9	743,542,862	13,849,098	0.58
Wolf	22.2	616,700,033	25,718,629	1.07	33.9	339,433,286	8,922,769	0.37

BP seq = base pair sequenced

Breadth = depth/BP seq

% = percent of whole genome

The regions obtained via GBS include 1,001,135 CpG regions which represents 3.98 ± 1.48% of the total number of CpGs (25 millions) in the canine genome. GBS-MeDIP include 149,275 ± 93,255 CpGs, 0.59 ± 0.29% of the total. The ratios (enrichment scores) between the number of observed CpGs and the expected from the reference genome were 1.37 and 2.16 for GBS and GBS-MeDIP, respectively, indicating an enrichment in CpGs for the GBS-MeDIP.

**Fig 1:**
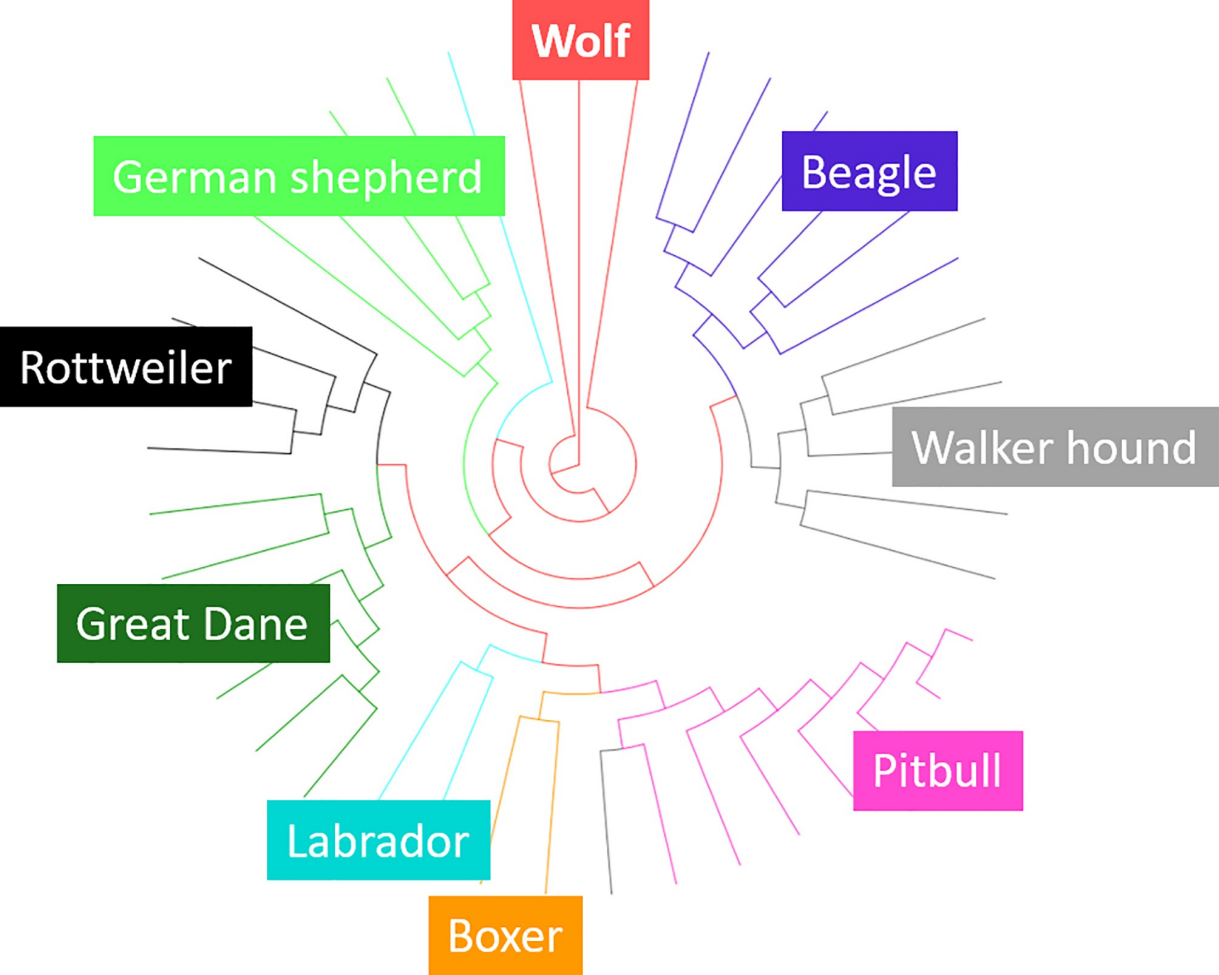
Neighbour-joining tree based on SNPs in the GBS reduced genome. Although only a reduced representation sample was used, individuals from the same species/breed group together, except two outliers (one Labrador (turquoise) and one walker hound (grey)).

A neighbor-joining tree was plotted based on 62,452 single nucleotide polymorphisms (SNPs) across all the 41 individuals from the GBS sequencing results. Although we only used a reduced genome, individuals from the same species/breed grouped together (except for two outliers), indicating that the GBS produced results representative of the genetic variability in each breed (Fig 1).

The number of SNPs in the GBS reduced genome that was fixed within species and breed groups was compared across groups. A total of 2479 SNPs with allele changes were found between the wolf and all dogs combined. The results from additional comparisons are presented in Table 2. For example, in wolf versus breed comparisons, boxer was the breed with greatest number of allele differences (2940 fixed SNPs) and walker hound the breed with least (745 SNPs) compared to wolves. When comparing the breeds, walker hound compared to pitbull terrier revealed the lowest number of allele changes (7 SNPs), while German shepherd dog and boxer differed in 1603 fixed SNPs.

**Table 2:** The number of differences in allele changes (SNPs fixed within species and breeds) between compared groups.

	Wolf	Beagle	Boxer	GSD	Great D.	Labrador	Pitbull	Rottw.	Walker
Wolf	-	1342	2940	1920	1374	1613	943	1779	745
Beagle		-	844	406	139	283	62	283	19
Boxer			-	1603	821	1177	313	1273	288
GSD				-	434	668	210	638	139
Great D.					-	316	68	295	32
Labrador						-	116	456	75
Pitbull							-	126	7
Rottw.								-	90
Walker									-

GSD: German shepherd dog

### Wolf-dog methylation differences

**Fig 2:**
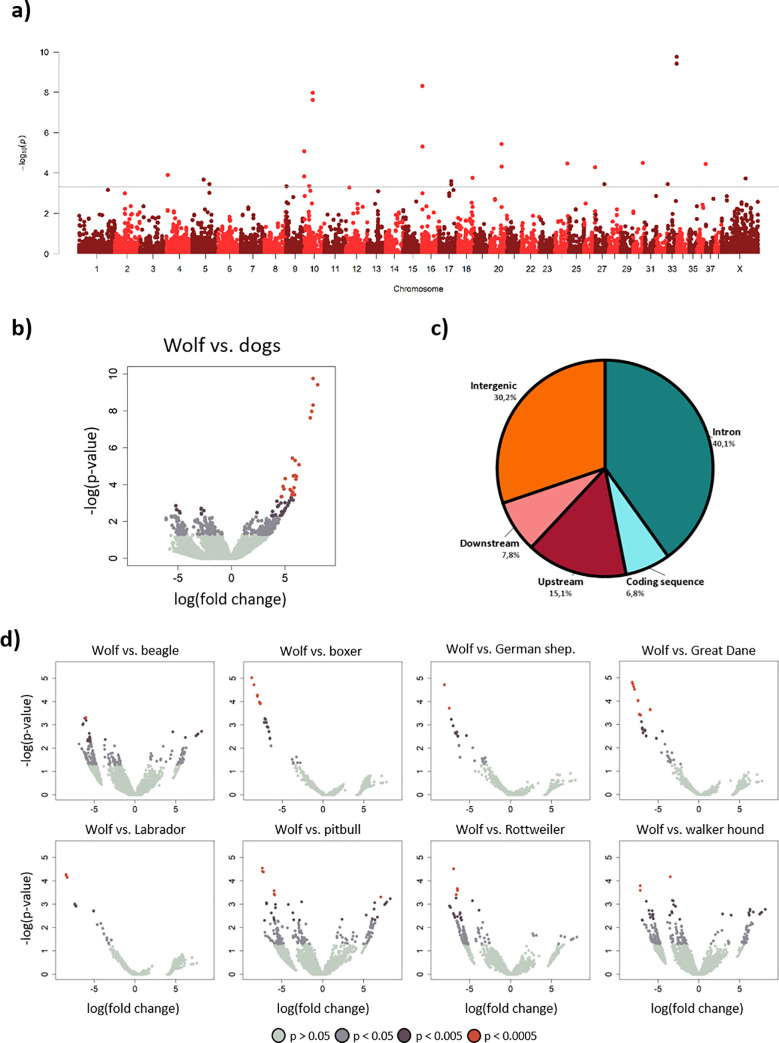
Differentially methylated regions (DMRs) between wolf and dog. a) Manhattan plot of DMRs between wolf and dogs. Line represents p<0.0005 cutoff. b) Volcano plot with DMRs for wolf and dogs. c) Pie chart of in what sequence variant DMRs p<0.005 are located in relation to the closest gene. d) Volcano plots with DMRs from analyses between wolf and each breed. For both a) and d), DMRs with negative log fold change are hypomethylated in the wolf and DMRs with positive log fold change are hypermethylated.

For the comparisons between wolves and dogs, only female individuals were included, since we only had brains from female wolves. The differentially methylated regions (DMRs) comparing wolf and dog are visualized in Manhattan and volcano plots in Fig 2A and 2B, respectively. In this reduced representation sample, there were 64 significant DMRs (p<0.0005) across 15 chromosomes between wolf and dog, located in, or close to, 11 unique genes (Table 3). All of these were hypermethylated in the wolf compared to dogs. The position of the DMRs relative to their closest genes is shown in Fig 2C. As can be seen, most DMRs are found in introns.

**Table 3:** List of genes with significant DMRs between wolf and dogs and wolf and breeds. Location is presented as chromosome:start-stop and adjacent regions have been merged. Strand is indicated by 1 or -1. Significant DMRs in intergenic regions are not included. P<0.0005 is considered significant.

Comp-arison	Location		Position	Gene (Ensmbl)	Symbol	Gene name
**Dogs**	4:3859701–3860000	-1	Intron	ENSCAFG00000011101	GPR137B	G protein-coupled receptor 137B
	5:38033301–38033800	1	Intron	ENSCAFG00000028975	HS3ST3B1	Sulfotransferase
	5:58084401–58084800	-1	Intron	ENSCAFG00000019477	MEGF6	Multiple EGF Like Domains 6
	9:860301–860500	1	Intron	ENSCAFG00000005719	TEPSIN	Adaptor related protein complex 4
	10:16973601–16973900	1	Coding seq.	ENSCAFG00000000673	PLXNB2	Plexin B2
	10:29677701–29678100	-1	Intron	ENSCAFG00000038553	-	*Uncharacterized*
	10:454201–454800	1	Intron	ENSCAFG00000000108	ESYT1	Extended synaptotagmin 1
	16:1003001–1003300	-1	Downstream	ENSCAFG00000034234	-	*Uncharacterized*
	17:40393501–40393800	1	Intron	ENSCAFG00000007987	SUCLG1	Succinate—CoA ligase [ADP/GDP-forming] subunit alpha
	20:39424401–39424800	1	Coding seq.	ENSCAFG00000011008	CAMKV	CaM kinase like vesicle
	24:42506201–42506501	-1	Intron	ENSCAFG00000012058	CTCFL	CCCTC-binding factor like
**Boxer**	1:93322101–93322600	1	5’ UTP	ENSCAFG00000002102	JAK2	Tyrosine-protein kinase
	1:93322101–93322600	-1	Upstream	ENSCAFG00000039255	-	*Uncharacterized*
	25:48720101–48720500	-1	Intron	ENSCAFG00000012433	PER2	Period circadian clock 2
**Great D**	7:67036001–67036140	1	Coding seq.	ENSCAFG00000018309	USP14	Ubiquitin specific peptidase 14
	7:67036001–67036140	1	Downstream	ENSCAFG00000018282	ROCK1	Rho-associated protein kinase
	8:986301–986700	-1	Intron	ENSCAFG00000010820	CATSPERB	Cation channel sperm associated auxiliary subunit beta
	14:20122501–20123000	1	Intron	ENSCAFG00000032379	PEG10	Paternally Expressed 10
	14:20122501–20123000	-1	Upstream	ENSCAFG00000002104	SGCE	Sarcoglycan epsilon
	30:39272601–39273000	-1	Coding seq.	ENSCAFG00000018073	PSTPIP1	Proline-serine-threonine phosphatase interacting protein 1
	36:18696701–18696800	-1	Intron	ENSCAFG00000013271	WIPF1	WAS/WASL Interacting Protein Family Member 1
**GSD**	9:2860101–2860300	-1	Intron	ENSCAFG00000005312	PGS1	CDP-diacylglycerol—glycerol-3-phosphate 3-phosphatidyltransferase
	9:2860101–2860300	1	Downstream	ENSCAFG00000005306	SOCS3	Suppressor of cytokine-signaling 3
	21:35980601–35981000	1	Downstream	ENSCAFG00000008082	RASSF10	Ras association domain family member 10
**Labrad.**	18:52431001–52431600	1	Downstream	ENSCAFG00000014309	RASGRP2	RAS guanyl releasing protein 2
**Rottwei.**	4:34785701–34786100	-1	Intron	ENSCAFG00000016100	SYT15/LOC489080	Synaptotagmin 15
	4:34785701–34786100	-1	Downstream	ENSCAFG00000016101	GPRIN2	G protein regulated inducer of neurite outgrowth 2
**Walker**	9:40807801–40808200	-1	Coding seq.	ENSCAFG00000018462	RNF135	Ring finger protein 135

GSD: German shepherd dog

DNA methylation was also compared between wolf and each of the eight breeds separately (Fig 2D). In contrast to the DMRs in the wolf-dog comparison, in the breed comparisons, a majority of the significant DMRs were hypomethylated in the wolf (represented by a negative log fold change). Across all breeds, there were in total 116 significant DMRs in 17 genes (Table 3). Interestingly, few DMRs overlap across the different comparisons between wolves and dogs.

The gene list from the first comparison, wolf versus all dogs, was further analyzed in GO analysis and pathway analyses. 183 genes (DMRs p<0.05) were used as input, of which 40 were annotated to a specific GO function and 65 assigned specific pathway. The GO analysis identified an enrichment for, among other things, regulation of nervous system development, synapse structure, and cell development. GO terms related to anatomical structures were also found (p<0.05; Table 4). However, none reached adjusted significance (false discovery rate, FDR). The pathway analysis identified two pathways that reached adjusted significance (FDR<0.05): 1) TNF signaling and 2) SHC-related events triggered by IGF1R. Both of these are signal transduction pathways. TNF signaling pathway is involved in, for example, cell growth and death and immune and inflammatory responses [50], and IGF1R signaling promotes cell growth and differentiation. Interestingly, IGF1 is a determinant of small size in dogs [51]. Other pathways that we found were related to, among others, developmental biology (axon guidance), immune response and metabolism (p<0.05) (Table 5). High level GO terms for genes from both wolf versus dog and wolf versus breed comparisons are presented in S2 Table. The genes are related to a wide array of processes, but noteworthy are genes involved in neurological processes, stress response, and reproduction. It is important to mention that due to the nature of these analyses, the pathways showing enrichment are biased towards genes that have been extensively investigated in the literature.

**Table 4:** Results from the gene ontology (GO) enrichment analysis for DMRs between wolf and dog. Go terms for biological process and molecular function with p-values <0.05 are presented. No Go terms for cellular component reached significance. Observed (O), expected (E), ratio O/E (R), p-value (p), and false discovery rate (FDR) are show for each term, as well as the contributing genes.

	GO term	Description	O	E	R	p	FDR	Genes
**Biological process**	GO:0007389	pattern specification process	5	1,0	5,1	<0,01	0,79	HOXD10, ACVRL1, HOXA11, HES4, GRHL3
	GO:0051960	regulation of nervous system development	6	1,7	3,6	0,01	0,79	AMIGO3, EFNA1, ULK4, HES4, GDI1, GRHL3
	GO:0009790	embryo development	7	2,3	3,1	0,01	0,79	HOXD10, EFNA1, ACVRL1, HOXA11, HES4, COL6A1, GRHL3
	GO:0050803	regulation of synapse structure or activity	3	0,5	6,2	0,01	1,00	CAMKV, AMIGO3, EFNA1
	GO:0022603	regulation of anatomical structure morphogenesis	6	2,0	3,0	0,01	1,00	EFNA1, ACVRL1, PDZD8, HOXA11, GDI1, GRHL3
	GO:0022406	membrane docking	2	0,2	10,3	0,02	1,00	EXOC6B, PDZD8
	GO:0060284	regulation of cell development	5	1,6	3,1	0,02	1,00	EFNA1, ULK4, HOXA11, HES4, GDI1
	GO:0009887	animal organ morphogenesis	6	2,3	2,7	0,02	1,00	HOXD10, EFNA1, ACVRL1, HOXA11, COL6A1, GRHL3
	GO:0051640	organelle localization	4	1,2	3,4	0,03	1,00	CCNB1, EXOC6B, PDZD8, NDC80
	GO:0048646	anatomical structure formation involved in morphogenesis	6	2,4	2,5	0,03	1,00	EFNA1, ACVRL1, HOXA11, HES4, COL6A1, GRHL3
**Molecular function**	GO:0016772	transferase activity, transferring phosphorus-containing groups	7	2,2	3,1	0,01	0,64	ULK4, CCNB1, SUCLG1, CAMKV, ADK, AK1, ACVRL1
	GO:0019838	growth factor binding	2	0,3	6,2	0,04	1,00	COL6A1, ACVRL1
	GO:0030234	enzyme regulator activity	5	2,0	2,5	0,05	1,00	CCNB1, CAST, ITIH1, GDI1, PHACTR3

**Table 5:** Results from the pathway analysis based on DMRs between wolf and dog. Reactome pathway name and description are presented. Pathway hierarchy represents the highest hierarchy branches for the pathways. P-value (p), false discovery rate (FDR), and contibuting genes are shown.

Pathway	Description	p	FDR	Pathway hierarchy	Genes
R-CFA-399954	Sema3A PAK dependent Axon repulsion	0,01	0,38	Developmental biology—axon guidance	PLXNA3
R-CFA-399955	SEMA3A-Plexin repulsion signaling by inhibiting Integrin adhesion	<0,01	0,25	Developmental biology—axon guidance	PLXNA3
R-CFA-399956	CRMPs in Sema3A signaling	0,01	0,38	Developmental biology—axon guidance	PLXNA3
R-CFA-203927	MicroRNA (miRNA) biogenesis	0,01	0,41	Gene expression—gene silencing	PRKRA
R-CFA-426486	Small interfering RNA (siRNA) biogenesis	0,02	0,43	Gene expression—gene silencing	PRKRA
R-CFA-1236973	Cross-presentation of particulate exogenous antigens (phagosomes)	0,02	0,43	Immune response—adaptive	ITGB5
R-CFA-1606322	ZBP1(DAI) mediated induction of type I IFNs	0,04	0,43	Immune response—innate	IKBKG
R-CFA-5684264	MAP3K8 (TPL2)-dependent MAPK1/3 activation	0,04	0,43	Immune response—innate	IKBKG
R-CFA-937039	IRAK1 recruits IKK complex	0,05	0,43	Immune response—innate	IKBKG
R-CFA-975144	IRAK1 recruits IKK complex upon TLR7/8 or 9 stimulation	0,05	0,43	Immune response—innate	IKBKG
R-CFA-5661270	Formation of xylulose-5-phosphate	0,01	0,30	Metabolism—carbohydrates	DCXR
R-CFA-1855167	Synthesis of pyrophosphates in the cytosol	0,03	0,43	Metabolism—inositol phosphate	IP6K1
R-CFA-1855191	Synthesis of IPs in the nucleus	<0,01	0,19	Metabolism—inositol phosphate	IP6K1
R-CFA-428157	Sphingolipid metabolism	0,05	0,43	Metabolism—lipids	CERS2, ESYT1, GLB1L
R-CFA-4755510	SUMOylation of immune response proteins	0,01	0,38	Metabolism of proteins	IKBKG
R-CFA-75893	TNF signaling	<0,01	0,04	Signal transduction—death receptor signaling	TRAF1, IKBKG, SHARPIN
R-CFA-2428933	SHC-related events triggered by IGF1R	<0,01	0,04	Signal transduction—IGF1R signaling	IGF2

### Methylation differences across dog groups

**Fig 3:**
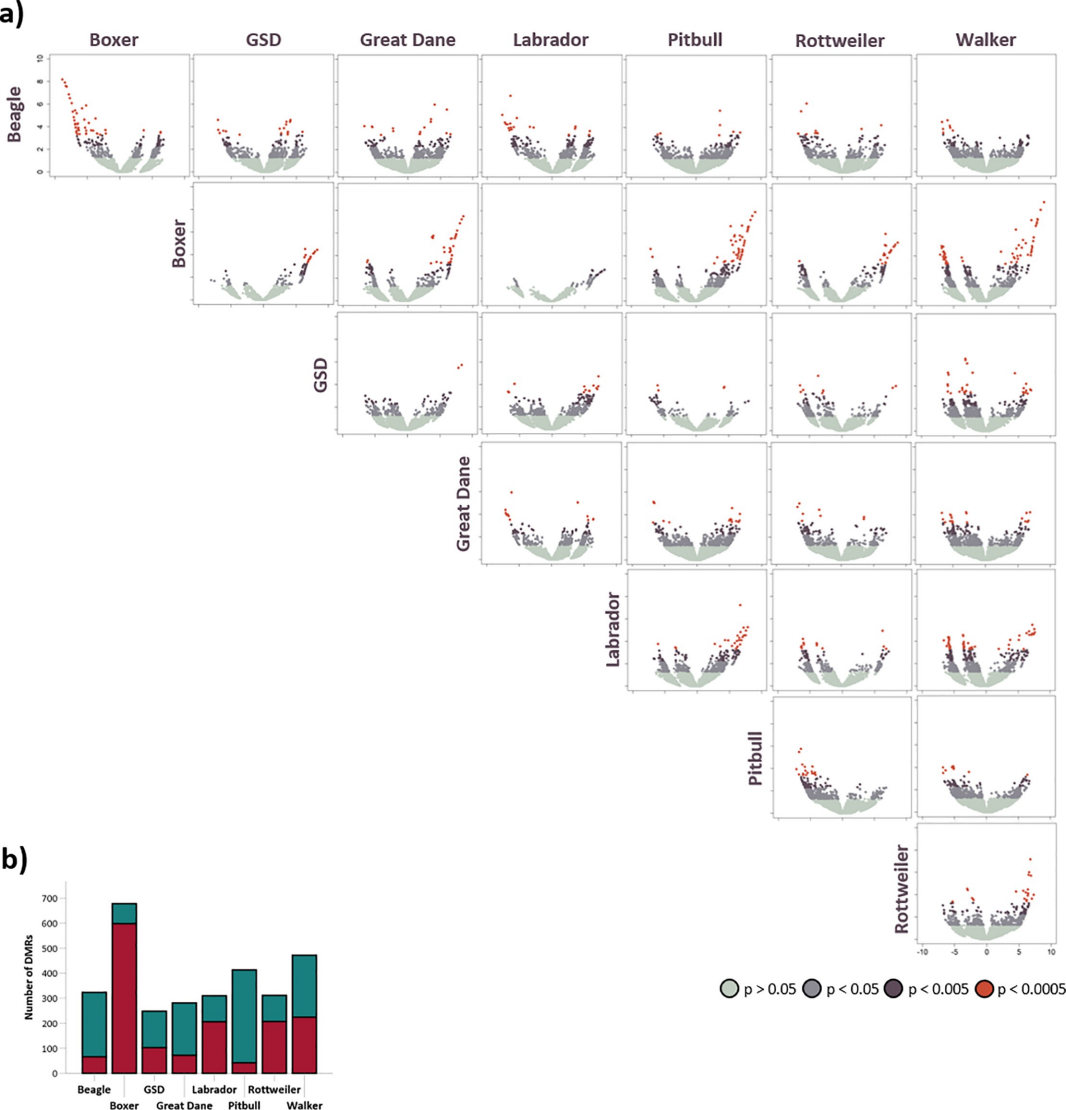
Differentially methylated regions (DMRs) between breeds. a) Volcano plot for each breed comparison where the vertical was compared to the horizontal (left to right). Thus, DMRs with negative log fold change are hypomethylated in the vertical and DMRs with positive log fold change are hypermethylated. b) The number of DMRs (p<0.05) for each breed from the breed comparisons. Colors indicate number of DMRs with positive (red) versus negative (blue) log fold change. (GSD: German shepherd dog).

Each breed was also compared to each of the other breeds. Volcano plots for each comparison are presented in Fig 3A. The boxer was the most divergent and had the largest number of DMRs that were hypermethylated compared to the other breeds. German shepherd dog had the least DMRs, and Beagle, Great Dane, and pitbull terrier were hypomethylated (Fig 3B). Together, the DMRs were located in 272 genes and a list of these is available in the S3 Table. High-level GO terms are presented in S4 Table. Noteworthy are genes involved in anatomical structures, behavior, neurotransmitter secretion, and stress response.

**Fig 4:**
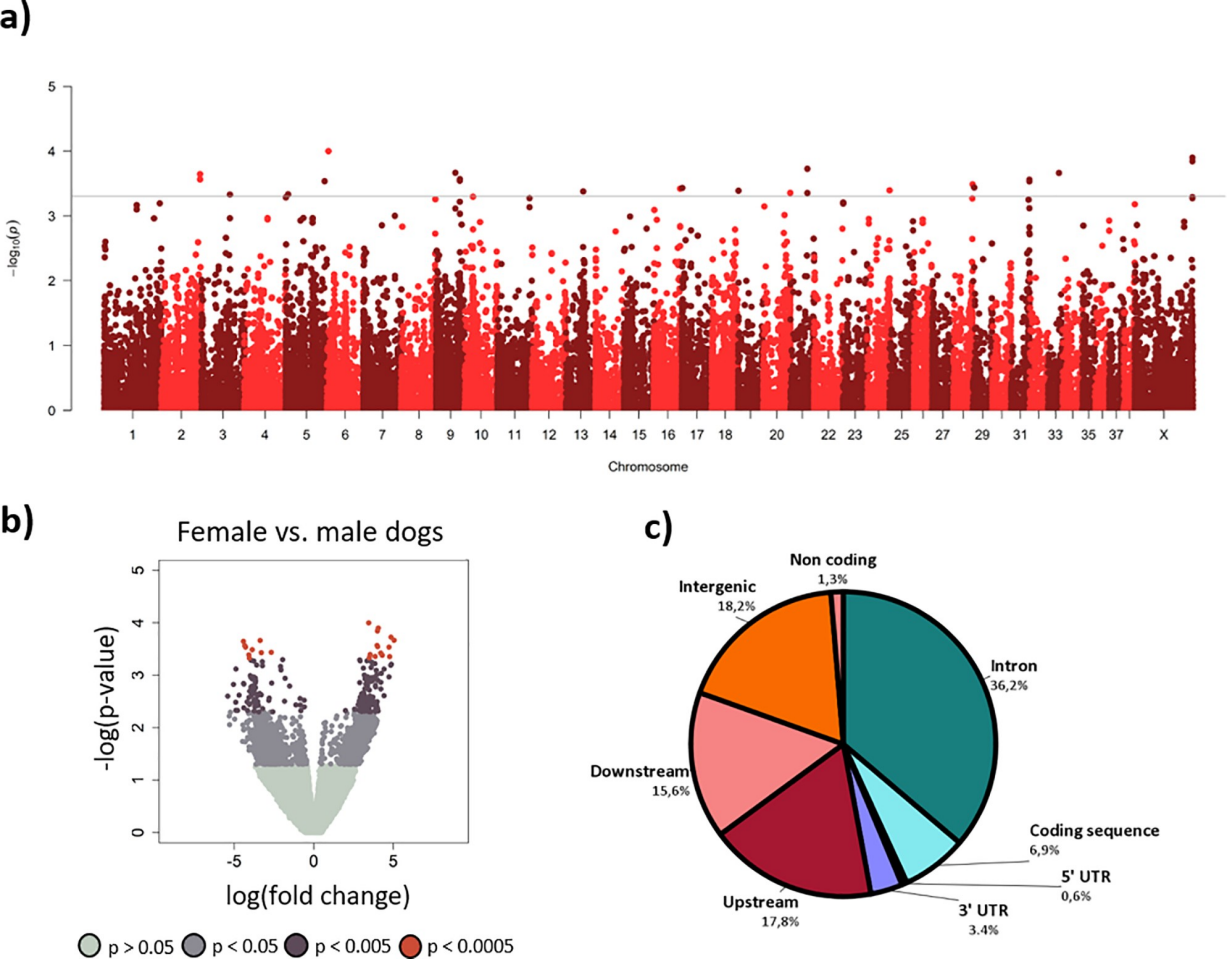
Differentially methylated regions (DMRs) between female and male dogs. a) Manhattan plot of DMRs between female and male dogs. Line represents p<0.0005 cutoff. b) Volcano plot with DMRs for female and male dogs. DMRs with negative log fold change are hypomethylated in females and DMRs with positive log fold change are hypermethylated. c) Pie chart of in what sequence variant DMRs p<0.005 are located in relation to the closest gene.

Additionally, female and male dogs were compared, and we found several differences. In the reduced representation sample, 75 DMRs differed between the sexes, situated across 17 chromosomes and located in, or close to, 21 unique genes (S5 Table). Females were more methylated than males at a majority of the DMRs (64%). The differences are visualized in a Manhattan plot (Fig 4A) and a volcano plot (Fig 4B). Most DMRs were found in introns followed by intergenic and upstream regions (Fig 4C). High level GO terms for groups of genes are presented in S6 Table. Although breeds were not balanced for sex, no overlaps of DMRs (p<0.005) were found between breed and sex analyses.

## Discussion

The role of epigenetic factors in evolution, domestication, and selection is receiving increasing attention and it has been suggested that epigenetics may play a greater role than previously assumed. In the present study, we explored differences in DNA methylation in the brain of domesticated dogs and their ancestor species, the grey wolf, and between breeds of dogs, which reflects a more recent selection. By utilizing a reduced fraction of the canine genome, we found distinct DNA methylation profiles in the brains of the wolf and dog as well as for the different dog breeds, suggesting that epigenetics has been important in the divergent selection during dog domestication and breed formation. Our results, however, cannot discriminate between true methylation alterations and potential confounding factors such as copy number variations in the regions showing methylation differences [52].

DNA methylation differences between ancestral and domesticated populations have been shown in previous studies comparing DNA methylation in blood and buccal samples of wolves and dogs [28, 29], and comparing domesticated chickens to their ancestor [18, 19]. Methylation differences have also been demonstrated between populations of Red Junglefowl selected for either high or low tameness over only five generations [53]. Although our present results do not allow any conclusions about specific causative effects, they strongly suggest that DNA-methylation is affecting or being affected by selection during dog domestication.

Because species-specific DNA methylation patterns have been shown to be stable across generations [18, 28], we can, based on our limited but very valuable biological material, compare species and patterns of DNA methylation related to the breeds. Our results show that the approach of combining genotype-by-sequencing and methylated DNA immunoprecipitation can be used in canines as a genotyping and epigenotyping method. GBS has been used previously to test domestication scenarios in chickens (*Gallus gallus*) [19] and in Lima beans (*Phaseolus lunatus L*.) [54], and the combined GBS-MeDIP has been used to study effects of different rearing conditions on chicken DNA methylation profiles in erythrocytes [37]. The reduced genome from the GBS procedure represented approx. 1% of the canine genome and combined with MeDIP it represented approx. 0.5%. Although only a small fraction of the genome was sequenced, the cladogram groups the individuals correctly according to group (wolf and breeds), similarly to previous whole-genome studies [22, 23]. Hence, in spite of the fact that occasional recognitions sites for the enzyme may differ between individuals due to sequence differences, the reduced fractions are sufficiently similar across the populations in order to produce a correct cladogram.

The methylated regions of the female wolves were compared to female dogs in two ways: 1) to the complete set of dogs and 2) to each of the eight breeds. In both types of analyses, we found several DMRs between wolves and dogs. However, different patterns emerged from the different analyses. In the comparison with all dogs, DMRs were hypermethylated in the wolves whereas the opposite was found when comparing the wolf to each of the breeds. Also, there were few overlaps in significant DMRs across the wolf-breed analyses. When we compare wolves to a pooled sample of different dog populations, we explore mainly epigenetic effects related to domestication, whereas when we compare wolves to each breed, we rather explore breed diversification. This implies that DNA methylation may be an important factor in the recent intense dog selection process, and this may perhaps be valid for speciation in general. This is further supported by our finding of substantial differences in methylation patterns between dog breeds. Previous studies have identified somatic DNA methylation differences as being important in speciation. Skinner, Gurerrero-Bosagna [55] found that epigenome differences between different species of Darwin’s finches were a better match than DNA sequence variation with respect to the evolutionary relationship. In line with this, Smith, Martin [56] found that the epigenome across and within darter populations, a stream fish of genus *Etheostomais sp*., were more diverse and changed faster than the genome, and that behavioral isolation of a population increased with DNA methylation differences. In chicken domestication and breed formation, CpG-related SNPs are reported to increase with genetic distance, which suggests an important role for DNA methylation in speciation [19].

Selection for behavior has been proposed as being the main driver in domestication (Trut et al., 2009; Agnvall et al., 2018), and genetic studies suggest that neurobiological processes have been targeted by selection during domestication (Saetre et al., 2004; Trut et al., 2009; vonHoldt et al., 2010; Axelsson et al., 2013; Li et al., 2014). Epigenetic factors are tissue specific [30] and the brain is the organ of interest for behavioral effects. Therefore, the tissue we have used to measure methylation differences is the brain. It is decidedly interesting that our results have highlighted genes and processes that may have been important for domestication and breed divergence. These include genes related to neurological processes, behavior, and synapse activity. Interestingly, Janowitz Koch, Clark [28] found genes related to the neurotransmitters GABA and glutamate to be differentially methylated between wolf and dog. This was, however, from blood samples. In the present study, using brain samples, we find several DMRs in genes related to neurotransmission between the domestic dog and their ancestor, giving further evidence that domestication has targeted neurological functions. For example, TEPSIN protein is part of the adaptor protein complex-4 that interacts with a glutamate receptor [57]; the expression of *CAMKV* is regulated by a glutamate receptor (*AMPA*) [58]; *PLXNB2* regulates GABAergic and glutamatergic synapse development [59]; *GRIN2B* is an effector in a pathway activated by, among others, neurotransmitters, and that leads to neurite outgrowth [60]; *JAK2* and *ROCK* are parts of pathways that regulate GABA expression [61].

We also identified differentially methylated genes between dog breeds that are interesting from a behavioral viewpoint, indicating that DNA methylation of these regions is important also for breed differences. For example, *SLC17A5* (sialin) transports neurotransmitters glutamate and aspartate [62], and *PTPRZ1*, that has been associated to schizophrenia. Mice overexpressing this gene show hyperactive behavior and have an altered glutamatergic, GABAergic and dopaminergic activity [63]. It has been shown that DNA methylation can affect behavior. In rats, maternal care affects methylation of the glucocorticoid receptor gene and alters stress reactivity [8]; in great tits, explorative behavior is affected by methylation level at a dopamine receptor gene [64], and in dogs, DNA methylation in the promoter region of the oxytocin receptor gene affects dog’s social behavior [10].

Wolf and dog breeds do not only differ in behavior, but also in morphological traits. Therefore, it is also of interest that we have identified DMRs in genes related to morphology.

From the pathway analysis, an *IGF1* signaling pathway was significant. The *IGF1* gene is involved in several prenatal and postnatal processes and it regulates imprinted genes, many of which control metabolism and growth [65]. The gene is a known determinant of small size in dogs [51] and has also been associated with dog anxiety [66]. In the GO analysis, the most relevant was ‘pattern specific process’. *Hox* genes, for example, are involved in forming the appendicular skeleton and mutant mice show dramatic phenotypic differences [67]. Interestingly, the dog breed pug shows typical vertebral anomalies consistent with altered *hox* genes [68]. Furthermore, we found DMRs in *PER2* gene in the wolf-boxer comparison. In humans, DNA methylation in this gene has been associated to obesity [69].

Most DMRs were found in intronic regions. It has been reported that DNA methylation of introns regulates gene expression. Anastasiadi, Esteve-Codina [70] found, across species, that the methylation level of the first intron was inversely linked with expression levels. Specifically, it has been suggested that neurological-related processes may be regulated by intronic DNA methylation [71]. DNA methylation can also affect splicing outcomes [72], and mutations in introns may affect splicing with consequences for RNA processing [73]. Interestingly, mutations in splicing sites have been associated with, for instance, human neurological diseases [74, 75]. The relevance of genetic and epigenetic changes of intronic and intergenic regions merits further investigation.

As expected, and in accordance with previous studies on other species [32, 33, 53], we also found sexually dimorphic DNA methylation profiles in dogs. DMRs are found both in autosomes and sex chromosomes. Female DMRs were more methylated than males which has previously been shown in, for example, human and rat brains [32]. There are behavioral differences between female and male dogs [e.g. 76–78], where epigenetic factors seem to be important. For example, the level of methylation of the oxytocin receptor gene has been reported to be linked to differences in the social behavior in male and female dogs [29]. Moreover, it has been shown that DNA methylation of specific regions are strongly associated with sex-specific gene expression [33], and environmental challenges may have sexually dimorphic effects on DNA methylation and, consequently, on gene expression and behavior [79].

Our study has a number of important limitations. Firstly, the samples were all obtained from *ad hoc* donated brains, so we can not be certain that they are truly representative of the different breeds. However, the phylogenetic analysis clearly showed that the samples clustered according to breed with respect to DNA-sequence variation, so we feel relatively confident in assuming that they should grossly represent the breeds. Secondly, the dogs were of different age, and it is well known that methylation patterns may change over life, as has been demonstrated in leucocytes [80]. However, the dynamic part of the methylome represents a relatively minor fraction of the methylation differences, so we believe that the majority of the DMR’s detected here are permanent, so called obligatory epigenetic variation that are considerably more stable over time. It is also to be expected that methylation in brain neurons are more stable than in leucocytes. Furthermore, there were no systematic age differences between the dogs from the different breeds. This also pertains to the third important caveat, and that is the fact that some epigenetic variation may occur due to environmental impact. Again, this will cause dynamic, or facultative, stochastic methylation differences and should logically not affect the present results to any major degree.

In conclusion, we show that the epigenome of the brain, in the form of DNA methylation pattern, differs substantially between the wolf and the domestic dog, and between different dog breeds. This tentatively suggests that epigenetic factors may have been, and still are, important mechanisms involved in dog domestication and dog-breed formation. Specifically, we highlight methylation differences in genes related to behavior and morphology. Although the causal effects of methylation differences between and within canines remains to be studied, these may alter gene expression and, thereby, phenotype. We hypothesize that these differences are involved in the great phenotypic variation found among dogs and future studies should further explore the impact of epigenetic factors in relation to genetic factors. We also show that the new method of combining genotype-by-sequencing and methylated DNA immunoprecipitation can be used as an efficient epigenotyping method in canines. Our results are useful to further our understanding of epigenetic involvement in evolution and speciation, and they add an intriguing dimension to dog breeding.

## Supporting information

S1 Table Detailed description of combined GBS-MeDIP method for canine samples. (DOCX)

S2 Table High level gene ontology categories from ShinyGO v0.50 for genes with DMRs from the wolf comparisons (wolf vs. Dogs and wolf vs. Breeds). (DOCX)

S3 Table List of genes with significant DMRs (p<0.0005) from the breed comparisons. Breeds Beagle (Bea), boxer (Box), German shepherd dog (Ger), Great Dane (Grea), Labrador retriever (Lab), pitbull terrier (Pit), Rottweiler (Rot) and walker hound (Wal) are all compared in the direction of first compared to second.
(DOCX)

S4 Table High level gene ontology categories from ShinyGO v0.50 for genes with DMRs from the breed comparisons. (DOCX)

S5 Table List of genes with differentially methylated regions between female and male dogs. Location is presented as chromosome:start-stop and adjacent regions have been merged. Strand is indicated by 1 or -1. Negative log fold change (FC) is less methylated in females and positive more methylated in females.
(DOCX)

S6 Table High level gene ontology categories from ShinyGO v0.50 for genes with DMRs from the comparison between female and male dogs. (DOCX)
