# Supplementary material for: DNA methylation in canine brains is related to domestication and dog-breed formation
Source: PLoS One. 2020 Oct 29;15(10):e0240787. doi: 10.1371/journal.pone.0240787 (PMC7595415; doi:10.1371/journal.pone.0240787)
Supplement: S1 Table — (DOCX) [file pone.0240787.s001.docx]

**S1 Table.** Detailed description of combined GBS-MeDIP method for canine samples.

| **Step** | **Procedure** |
| --- | --- |
| **Cleavage:** | 200 ng of DNA was denatured and used in the cleavage reaction with 1 µl *PSTI*, 3.5 µl buffer 10x and completed with water to a total volume of 35 µl. Incubation was performed at 37° C for 16 h and 85° C for 20 min. |
| **Ligation:** | 16 µl (200 ng) digested DNA was added to 6 µl of adapters (0.6 ng/µl, barcode + common) and incubated on a plate shaker for 15 min at room temperature. A binding mix of 1 µl T4DNA ligase (400U), 3 µl of 10XT4DNA buffer and 4 µl water was mixed with the DNA + adapters and incubated in Thermocycler at 22° C for 2 h and 65° C for 30 min. Samples were then pooled and purified using QIAquick PCR purification according to manufacturer’s protocol. In order to maximize yield, elution was done twice using 30 µl of elution buffer. Part of the purified ligated DNA was thereafter used as the GBS library and the rest as an input for the MeDIP. |
| **GBS library:** | 60 ng of DNA from the previous step was used in polymerase chain reaction (PCR) to amplify the ligated fragments. The PCR program in the Thermocycler was as follows: 72° C for 5 min, 98° C for 30 s, (98° C for 10 s, 65° C 30 s, 72° C 30 s) x 18 cycles, 72° C for 5 min, 4° C forever. The PCR product was purified using Agencourt AMPURE XP using manufacturers protocol. |
| **MeDIP:** | The optimized protocol from Carlos & Jensen (2016) was used with few modifications to further optimize it for the combined GBS-MeDip procedure. First, the ligated DNA sample was dried in SpeedVac and resuspended in 70 µl water. 330 µl TE buffer was added and the solution was denatured. 100 µl of 5x IP buffer and 10 µg (5 µl) of anti-methylcytosine antibody was added and the DNA-antibody mixture was incubated at 4° C overnight on a slowly rotating platform. 160 µl of agarose beads was centrifuged at 6000 rpm for 2 min at 4° C and the supernatant was discarded. The DNA-antibody mixture was added to the beads and incubated 2 h at 4° C on a slowly rotating platform. The reaction was centrifuged at 6000 rpm for 2 min at 4° C and the supernatant was discarded. The beads were washed three times repeating the following steps: 1 ml of 1x IP buffer was added, the mixture was incubated 5 min at 4° C on a slowly rotating platform followed by centrifugation at 6000 rpm for 2 min at 4° C and discarding of supernatant. The beads were then resuspended in 210 µl of digestion buffer, 20 µl proteinase K (20 mg/ml) was added and the mixture incubated 2 h at 55° C on a slowly rotating platform. The reaction was filtered with a Pierce spin-filtering column at 17,000 g for 30 s and to the flow through, 3 µl glycogen (5 mg/ml) was added. 20 µl cold 5 M NaCl and 750 µl cold 80 % ethanol was added and for the DNA to precipitate, the reaction was incubated for 30 min on at room temperature followed by centrifugation at 12,000 rpm for 30 min at 4° C. The supernatant was removed. To wash the pellet, 1 ml of 70 % ethanol was added, and the sample was vortexed, rotated at room temperature for 5 min and centrifuged at 12,000 rpm for 30 min at 4° C. The sample was dried at 50° C for 5 min, resuspended in 30 µl of water and incubated at 50° C for 5 min. |
| **GBS-MeDIP library:** | 50 ng of DNA from the MeDIP procedure was then amplified and the PCR program in the Thermocycler was as follows: 72° C for 5 min, 98° C for 30 s, (98° C for 10 s, 65° C 30 s, 72° C 30 s) x 23 cycles, 72° C for 5 min, 4° C forever. The PCR product was purified using Agencourt AMPURE XP. |
