# Supplementary material for: DNA methylation in canine brains is related to domestication and dog-breed formation
Source: PLoS One. 2020 Oct 29;15(10):e0240787. doi: 10.1371/journal.pone.0240787 (PMC7595415; doi:10.1371/journal.pone.0240787)
Supplement: S2 Table — (DOCX) [file pone.0240787.s002.docx]

| **S2 Table.** High level gene ontology categories from ShinyGO v0.50 for genes with DMRs from the wolf  comparisons (wolf vs. Dogs and wolf vs. Breeds). | |
| --- | --- |
| **High level GO category** | **Genes** |
| Anatomical structure development | ENSCAFG00000000673 ENSCAFG00000002102 ENSCAFG00000010820 ENSCAFG00000005306 ENSCAFG00000018282 |
| Anatomical structure formation involved in morphogenesis | ENSCAFG00000000673 ENSCAFG00000018282 |
| Anatomical structure morphogenesis | ENSCAFG00000000673 ENSCAFG00000005306 ENSCAFG00000018282 |
| Biological adhesion | ENSCAFG00000000673 ENSCAFG00000002102 ENSCAFG00000018282 |
| Biosynthetic process | ENSCAFG00000005312 ENSCAFG00000012058 |
| Catabolic process | ENSCAFG00000018282 ENSCAFG00000018309 |
| Cell adhesion | ENSCAFG00000000673 ENSCAFG00000002102 ENSCAFG00000018282 |
| Cell motility | ENSCAFG00000002102 ENSCAFG00000000673 ENSCAFG00000018282 |
| Cell proliferation | ENSCAFG00000002102 ENSCAFG00000000673 |
| Cellular component organization | ENSCAFG00000000673 ENSCAFG00000002104 ENSCAFG00000016100 ENSCAFG00000002102 ENSCAFG00000012058 ENSCAFG00000013271 ENSCAFG00000018282 |
| Cellular component organization or biogenesis | ENSCAFG00000000673 ENSCAFG00000002104 ENSCAFG00000016100 ENSCAFG00000002102 ENSCAFG00000012058 ENSCAFG00000013271 ENSCAFG00000018282 |
| Cellular localization | ENSCAFG00000016100 ENSCAFG00000000108 ENSCAFG00000018282 |
| Developmental process | ENSCAFG00000000673 ENSCAFG00000002102 ENSCAFG00000010820 ENSCAFG00000005306 ENSCAFG00000018282 |
| Developmental process involved in reproduction | ENSCAFG00000010820 ENSCAFG00000005306 |
| Establishment of localization | ENSCAFG00000016100 ENSCAFG00000000108 ENSCAFG00000018282 |
| Immune response | ENSCAFG00000002102 ENSCAFG00000018462 |
| Immune system process | ENSCAFG00000002102 ENSCAFG00000018282 ENSCAFG00000018462 |
| Localization | ENSCAFG00000002102 ENSCAFG00000016100 ENSCAFG00000000108 ENSCAFG00000000673 ENSCAFG00000018282 |
| Localization of cell | ENSCAFG00000002102 ENSCAFG00000000673 ENSCAFG00000018282 |
| Locomotion | ENSCAFG00000002102 ENSCAFG00000000673 ENSCAFG00000018282 ENSCAFG00000018309 |
| Macromolecule localization | ENSCAFG00000000108 ENSCAFG00000018282 |
| Multicellular organism reproduction | ENSCAFG00000010820 ENSCAFG00000012058 |
| Multicellular organismal reproductive process | ENSCAFG00000010820 ENSCAFG00000012058 |
| Multi-organism process | ENSCAFG00000010820 ENSCAFG00000012058 ENSCAFG00000013271 |
| Multi-organism reproductive process | ENSCAFG00000010820 ENSCAFG00000012058 |
| Negative regulation of biological process | ENSCAFG00000000673 ENSCAFG00000005306 ENSCAFG00000018282 ENSCAFG00000018309 |
| Positive regulation of biological process | ENSCAFG00000000673 ENSCAFG00000002102 ENSCAFG00000005306 ENSCAFG00000012058 ENSCAFG00000018282 ENSCAFG00000018462 |
| Regulation of biological quality | ENSCAFG00000000673 ENSCAFG00000002102 c |
| Regulation of cell adhesion | ENSCAFG00000000673 ENSCAFG00000002102 ENSCAFG00000018282 |
| Regulation of developmental process | ENSCAFG00000000673 ENSCAFG00000005306 ENSCAFG00000018282 |
| Regulation of localization | ENSCAFG00000016100 ENSCAFG00000000673 ENSCAFG00000018282 |
| Regulation of locomotion | ENSCAFG00000000673 ENSCAFG00000018309 |
| Regulation of metabolic process | ENSCAFG00000000673 ENSCAFG00000005306 ENSCAFG00000012058 ENSCAFG00000018282 ENSCAFG00000018309 |
| Regulation of molecular function | ENSCAFG00000000673 ENSCAFG00000014309 ENSCAFG00000018282 ENSCAFG00000018309 |
| Regulation of multicellular organismal process | ENSCAFG00000000673 ENSCAFG00000018282 ENSCAFG00000018462 |
| Regulation of response to stimulus | ENSCAFG00000002102 ENSCAFG00000005306 ENSCAFG00000018309 ENSCAFG00000018462 |
| Reproduction | ENSCAFG00000010820 ENSCAFG00000005306 ENSCAFG00000012058 |
| Reproductive process | ENSCAFG00000010820 ENSCAFG00000005306 ENSCAFG00000012058 |
| Response to chemical | ENSCAFG00000002102 ENSCAFG00000005306 ENSCAFG00000014309 ENSCAFG00000018309 |
| Response to endogenous stimulus | ENSCAFG00000002102 ENSCAFG00000005306 |
| Response to external stimulus | ENSCAFG00000002102 ENSCAFG00000005306 ENSCAFG00000013271 ENSCAFG00000018309 |
| Response to stress | ENSCAFG00000002102 ENSCAFG00000005306 ENSCAFG00000018309 ENSCAFG00000018462 |
| Sexual reproduction | ENSCAFG00000010820 ENSCAFG00000012058 |
| Signaling | ENSCAFG00000002102 ENSCAFG00000011008 ENSCAFG00000016100 ENSCAFG00000000673 ENSCAFG00000005306 ENSCAFG00000008082 ENSCAFG00000014309 ENSCAFG00000018282 ENSCAFG00000018309 |
|  |  |
