# Supplementary material for: DNA methylation in canine brains is related to domestication and dog-breed formation
Source: PLoS One. 2020 Oct 29;15(10):e0240787. doi: 10.1371/journal.pone.0240787 (PMC7595415; doi:10.1371/journal.pone.0240787)
Supplement: S3 Table — Breeds Beagle (Bea), boxer (Box), German shepherd dog (Ger), Great Dane (Grea), Labrador retriever (Lab), pitbull terrier (Pit), Rottweiler (Rot) and walker hound (Wal) are all compared in the direction of first compared to second. (DOCX) [file pone.0240787.s003.docx]

**S3 Table.** List of genes with significant DMRs (p<0.0005) from the breed comparisons. Breeds Beagle (Bea), boxer (Box), German shepherd dog (Ger), Great Dane (Grea), Labrador retriever (Lab), pitbull terrier (Pit), Rottweiler (Rot) and walker hound (Wal) are all compared in the direction of first compared to second.

| **Comparison** | **LogFC** | **Position of DMR** | **Gene (Ensembl)** | **Symbol** | | **Gene name - symbol - ortholog** | |
| --- | --- | --- | --- | --- | --- | --- | --- |
| BeaBox | -7,25 | Intron | ENSCAFG00000007662 | ACSL1 | | Acyl-CoA synthetase long chain family member 1;ACSL1 | |
| BeaBox | -5,83 | Intron | ENSCAFG00000017595 | ADPRM | | ADP-ribose/CDP-alcohol diphosphatase, manganese dependent;ADPRM | |
| BeaBox | -6,82 | Upstream | ENSCAFG00000024030 | CD1C | | CD1c molecule;CD1C | |
| BeaBox | -6,20 | Upstream | ENSCAFG00000018608 | C7 | | Complement C7;C7 | |
| BeaBox | 6,28 | Intron | ENSCAFG00000014992 | FMO3 | | Dimethylaniline monooxygenase [N-oxide-forming] 3;FMO3 | |
| BeaBox | -6,37 | Upstream | ENSCAFG00000030240 | DUSP4 | | Dual specificity protein phosphatase;DUSP4 | |
| BeaBox | -5,20 | Intron | ENSCAFG00000024578 | GPR182 | | G protein-coupled receptor 182;GPR182 | |
| BeaBox | -7,01 | Upstream | ENSCAFG00000019927 | LAMC3 | | Laminin subunit gamma 3;LAMC3 | |
| BeaBox | -3,48 | Intron | ENSCAFG00000031536 | LDLRAD4 | | Low density lipoprotein receptor class A domain containing 4;LDLRAD4 | |
| BeaBox | 6,28 | Intron | ENSCAFG00000014998 | MROH9 | | Maestro heat like repeat family member 9;MROH9 | |
| BeaBox | -6,78 | Coding sequence | ENSCAFG00000014350 | MEF2B | | Myocyte enhancer factor 2B;MEF2B | |
| BeaBox | -3,51 | Intron | ENSCAFG00000029664 | NUDT19 | | Nudix hydrolase 19;NUDT19 | |
| BeaBox | -5,83 | Upstream | ENSCAFG00000032704 | SCO1 | | SCO1, cytochrome c oxidase assembly protein;SCO1 | |
| BeaBox | -5,20 | Intron | ENSCAFG00000000163 | STAT6 | | Signal transducer and activator of transcription;STAT6 | |
| BeaBox | -4,10 | Downstream | ENSCAFG00000019382 | SGTA | | Small glutamine rich tetratricopeptide repeat containing alpha;SGTA | |
| BeaBox | -5,19 | Intron | ENSCAFG00000010665 | SLC16A14 | | Solute carrier family 16 member 14;SLC16A14 | |
| BeaBox | -6,92 | Upstream | ENSCAFG00000002658 | SLC17A5 | | Solute carrier family 17 member 5;SLC17A5 | |
| BeaBox | -4,10 | Coding sequence | ENSCAFG00000031660 | SLC39A3 | | Solute carrier family 39 member 3;SLC39A3 | |
| BeaBox | -6,71 | Intron | ENSCAFG00000005719 | TEPSIN | | TEPSIN, adaptor related protein complex 4 accessory protein;TEPSIN | |
| BeaBox | -5,21 | Downstream | ENSCAFG00000023924 | CD151 | | Tetraspanin;CD151 | |
| BeaBox | -6,47 | Coding sequence | ENSCAFG00000025247 | TMEM121B | | Transmembrane protein 121B;TMEM121B | |
| BeaBox | -5,35 | Intron | ENSCAFG00000018119 | TCOF1 | | Treacle ribosome biogenesis factor 1;TCOF1 | |
| BeaBox | -3,05 | Intron | ENSCAFG00000016100 | - | | Uncharacterized protein;LOC489080 | |
| BeaBox | -7,09 | Downstream | ENSCAFG00000003500 | NOBOX | | Uncharacterized protein;NOBOX | |
| BeaBox | -4,42 | Intron | ENSCAFG00000012668 | - | | Uncharacterized protein;unassigned | |
| BeaBox | -3,05 | Intron | ENSCAFG00000023889 | - | | Uncharacterized protein;unassigned | |
| BeaBox | -6,37 | Intron | ENSCAFG00000025023 | - | | Uncharacterized protein;unassigned | |
| BeaBox | -6,37 | Downstream | ENSCAFG00000030880 | - | | Uncharacterized protein;unassigned | |
| BeaBox | -6,69 | Upstream | ENSCAFG00000031827 | - | | Uncharacterized protein;unassigned | |
| BeaBox | -6,69 | Intron | ENSCAFG00000032488 | - | | Uncharacterized protein;unassigned | |
| BeaBox | -8,33 | Intron | ENSCAFG00000003354 | VWC2 | | von Willebrand factor C domain containing 2;VWC2 | |
| BeaBox | -6,31 | Non coding | ENSCAFG00000035737 | - | |  | |
| BeaBox | -6,20 | Upstream | ENSCAFG00000036794 | - | |  | |
| BeaBox | 3,68 | Intron | ENSCAFG00000038613 | - | |  | |
| BeaBox | -6,69 | Upstream | ENSCAFG00000039645 | - | |  | |
| BeaGer | 5,95 | Intron | ENSCAFG00000023431 | ADGRB3 | | Adhesion G protein-coupled receptor B3;ADGRB3 | |
| BeaGer | -7,05 | Downstream | ENSCAFG00000023569 | ANKRD60 | | Ankyrin repeat domain 60;ANKRD60 | |
| BeaGer | -6,80 | Upstream | ENSCAFG00000008687 | CCT8 | | Chaperonin containing TCP1 subunit 8;CCT8 | |
| BeaGer | 4,10 | Intron | ENSCAFG00000016305 | GPHN | | Gephyrin;GPHN | |
| BeaGer | -6,24 | Intron | ENSCAFG00000016038 | LDB3 | | LIM domain binding 3;LDB3 | |
| BeaGer | -7,01 | Downstream | ENSCAFG00000019730 | SLC25A33 | | Solute carrier family 25 member 33;SLC25A33 | |
| BeaGer | -7,01 | Upstream | ENSCAFG00000019733 | TMEM201 | | Transmembrane protein 201;TMEM201 | |
| BeaGer | 3,71 | Downstream | ENSCAFG00000017721 | - | | Uncharacterized protein;unassigned | |
| BeaGer | -6,24 | Intron | ENSCAFG00000015966 | WAPL | | WAPL cohesin release factor;WAPL | |
| BeaGer | -5,82 | Intron | ENSCAFG00000007601 | ZNF536 | | Zinc finger protein 536;ZNF536 | |
| BeaGer | 2,45 | Intron | ENSCAFG00000036828 | - | |  | |
| BeaGrea | 3,53 | Intron | ENSCAFG00000009031 | CMYA5 | | Cardiomyopathy associated 5;CMYA5 | |
| BeaGrea | 5,98 | Intron | ENSCAFG00000001907 | CORIN | | Corin, serine peptidase;CORIN | |
| BeaGrea | 5,93 | Intron | ENSCAFG00000024839 | KIF21B | | Kinesin family member 21B;KIF21B | |
| BeaGrea | -4,16 | Intron | ENSCAFG00000018073 | PSTPIP1 | | Proline-serine-threonine phosphatase interacting protein 1;PSTPIP1 | |
| BeaGrea | -5,56 | Downstream | ENSCAFG00000018282 | ROCK1 | | Rho-associated protein kinase;ROCK1 | |
| BeaGrea | 4,09 | Downstream | ENSCAFG00000029188 | SCAMP4 | | Secretory carrier-associated membrane protein;SCAMP4 | |
| BeaGrea | -5,56 | Intron | ENSCAFG00000018309 | USP14 | | Ubiquitin specific peptidase 14;USP14 | |
| BeaGrea | 6,54 | Upstream | ENSCAFG00000031942 | - | |  | |
| BeaLab | -6,88 | Intron | ENSCAFG00000010243 | ARL3 | | ADP ribosylation factor like GTPase 3;ARL3 | |
| BeaLab | -5,91 | Upstream | ENSCAFG00000007614 | CCNE1 | | Cyclin E1;CCNE1 | |
| BeaLab | -3,42 | Downstream | ENSCAFG00000007870 | DCLK2 | | Doublecortin like kinase 2;DCLK2 | |
| BeaLab | -6,41 | Upstream | ENSCAFG00000010688 | - | | Fibroblast growth factor;unassigned | |
| BeaLab | -6,34 | Coding sequence | ENSCAFG00000019374 | GABRD | | Gamma-aminobutyric acid type A receptor delta subunit;GABRD | |
| BeaLab | -7,67 | Intron | ENSCAFG00000003228 | PXDN | | Peroxidasin;PXDN | |
| BeaLab | -7,27 | Downstream | ENSCAFG00000014309 | RASGRP2 | | RAS guanyl releasing protein 2;RASGRP2 | |
| BeaLab | -6,58 | Intron | ENSCAFG00000032121 | RBM38 | | RNA binding motif protein 38;RBM38 | |
| BeaLab | 3,57 | Intron | ENSCAFG00000013809 | - | | Uncharacterized protein;unassigned | |
| BeaLab | -5,44 | Coding sequence | ENSCAFG00000031745 | - | | Uncharacterized protein;unassigned | |
| BeaPit | -5,95 | Intron | ENSCAFG00000003882 | DDO | | D-aspartate oxidase;DDO | |
| BeaPit | 3,54 | Intron | ENSCAFG00000015753 | SFTPD | | Surfactant protein D;SFTPD | |
| BeaPit | 6,76 | Intron | ENSCAFG00000029547 | - | | Uncharacterized protein;unassigned | |
| BeaPit | 6,76 | Intron | ENSCAFG00000031797 | - | | Uncharacterized protein;unassigned | |
| BeaRot | -5,49 | Coding sequence | ENSCAFG00000006532 | DCHS1 | | Dachsous cadherin-related 1;DCHS1 | |
| BeaRot | -5,47 | Downstream | ENSCAFG00000016101 | GPRIN2 | | G protein regulated inducer of neurite outgrowth 2;GPRIN2 | |
| BeaRot | -3,80 | Coding sequence | ENSCAFG00000006686 | MFHAS1 | | Malignant fibrous histiocytoma amplified sequence 1;MFHAS1 | |
| BeaRot | -3,75 | Intron | ENSCAFG00000019602 | PLEKHG5 | | Pleckstrin homology and RhoGEF domain containing G5;PLEKHG5 | |
| BeaRot | -6,77 | Intron | ENSCAFG00000009210 | R3HCC1 | | R3H domain and coiled-coil containing 1;R3HCC1 | |
| BeaRot | -5,49 | Upstream | ENSCAFG00000026406 | RF00001 | |  | |
| BeaWal | -6,02 | Downstream | ENSCAFG00000000620 | PPP6R2 | | Protein phosphatase 6 regulatory subunit 2;PPP6R2 | |
| BeaWal | -5,20 | Upstream | ENSCAFG00000012704 | RBBP8NL | | RBBP8 N-terminal like;RBBP8NL | |
| BeaWal | -6,72 | Intron | ENSCAFG00000018462 | RNF135 | | Ring finger protein 135;RNF135 | |
| BeaWal | -6,02 | Downstream | ENSCAFG00000000612 | SBF1 | | SET binding factor 1;SBF1 | |
| BeaWal | -6,69 | Intron | ENSCAFG00000012301 | TNS3 | | Tensin 3;TNS3 | |
| BeaWal | -6,02 | Upstream | ENSCAFG00000030366 | - | | Uncharacterized protein;unassigned | |
| BeaWal | -6,02 | Upstream | ENSCAFG00000033291 | - | |  | |
| BoxGer | 7,16 | Downstream | ENSCAFG00000017507 | DOCK6 | | Dedicator of cytokinesis 6;DOCK6 | |
| BoxGer | 6,46 | Upstream | ENSCAFG00000005811 | FMNL2 | | Formin like 2;FMNL2 | |
| BoxGer | 6,29 | Intron | ENSCAFG00000024578 | GPR182 | | G protein-coupled receptor 182;GPR182 | |
| BoxGer | 7,05 | Intron | ENSCAFG00000024839 | KIF21B | | Kinesin family member 21B;KIF21B | |
| BoxGer | 7,16 | Coding sequence | ENSCAFG00000017366 | KANK2 | | KN motif and ankyrin repeat domains 2;KANK2 | |
| BoxGer | 6,98 | Intron | ENSCAFG00000012433 | PER2 | | Period circadian regulator 2;PER2 | |
| BoxGer | 6,29 | Intron | ENSCAFG00000000163 | STAT6 | | Signal transducer and activator of transcription;STAT6 | |
| BoxGer | 7,22 | 5' UTR | ENSCAFG00000002102 | JAK2 | | Tyrosine-protein kinase;JAK2 | |
| BoxGer | 8,26 | Intron | ENSCAFG00000003354 | VWC2 | | von Willebrand factor C domain containing 2;VWC2 | |
| BoxGer | 7,16 | Upstream | ENSCAFG00000028518 | - | |  | |
| BoxGer | 7,22 | Upstream | ENSCAFG00000039255 | - | |  | |
| BoxGrea | 6,55 | Intron | ENSCAFG00000017595 | ADPRM | | ADP-ribose/CDP-alcohol diphosphatase, manganese dependent;ADPRM | |
| BoxGrea | 6,98 | Upstream | ENSCAFG00000019412 | CASKIN1 | | CASK interacting protein 1;CASKIN1 | |
| BoxGrea | 6,57 | Downstream | ENSCAFG00000032715 | C7H1orf53 | | Chromosome 1 open reading frame 53;C7H1orf53 | |
| BoxGrea | 5,52 | Intron | ENSCAFG00000031536 | LDLRAD4 | | Low density lipoprotein receptor class A domain containing 4;LDLRAD4 | |
| BoxGrea | 6,57 | Coding sequence | ENSCAFG00000014350 | MEF2B | | Myocyte enhancer factor 2B;MEF2B | |
| BoxGrea | 6,49 | Intron | ENSCAFG00000019602 | PLEKHG5 | | Pleckstrin homology and RhoGEF domain containing G5;PLEKHG5 | |
| BoxGrea | 6,55 | Upstream | ENSCAFG00000032704 | SCO1 | | SCO1, cytochrome c oxidase assembly protein;SCO1 | |
| BoxGrea | 6,65 | Intron | ENSCAFG00000010665 | SLC16A14 | | Solute carrier family 16 member 14;SLC16A14 | |
| BoxGrea | 6,73 | Intron | ENSCAFG00000018119 | TCOF1 | | Treacle ribosome biogenesis factor 1;TCOF1 | |
| BoxGrea | 6,75 | Downstream | ENSCAFG00000003500 | NOBOX | | Uncharacterized protein;NOBOX | |
| BoxGrea | 6,43 | Intron | ENSCAFG00000012668 | - | | Uncharacterized protein;unassigned | |
| BoxGrea | -6,30 | Intron | ENSCAFG00000013632 | IL1RAPL1 | | Uncharacterized protein;unassigned | |
| BoxPit | 5,84 | Intron | ENSCAFG00000016016 | ACADVL | | Acyl-CoA dehydrogenase very long chain;ACADVL | |
| BoxPit | 7,45 | Intron | ENSCAFG00000007662 | ACSL1 | | Acyl-CoA synthetase long chain family member 1;ACSL1 | |
| BoxPit | 5,47 | Upstream | ENSCAFG00000024030 | CD1C | | CD1c molecule;CD1C | |
| BoxPit | 6,43 | Upstream | ENSCAFG00000016708 | DGKQ | | Diacylglycerol kinase;DGKQ | |
| BoxPit | 6,55 | Upstream | ENSCAFG00000030240 | DUSP4 | | Dual specificity protein phosphatase;DUSP4 | |
| BoxPit | 6,15 | Upstream | ENSCAFG00000019927 | LAMC3 | | Laminin subunit gamma 3;LAMC3 | |
| BoxPit | 6,55 | 5' UTR | ENSCAFG00000006686 | MFHAS1 | | Malignant fibrous histiocytoma amplified sequence 1;MFHAS1 | |
| BoxPit | 5,84 | Downstream | ENSCAFG00000016292 | NLGN2 | | Neuroligin 2;NLGN2 | |
| BoxPit | 6,75 | Intron | ENSCAFG00000029664 | NUDT19 | | Nudix hydrolase 19;NUDT19 | |
| BoxPit | 7,10 | Intron | ENSCAFG00000032520 | PTPRN2 | | Protein tyrosine phosphatase, receptor type N2;PTPRN2 | |
| BoxPit | 5,84 | Upstream | ENSCAFG00000016297 | SPEM2 | | SPEM family member 2;SPEM2 | |
| BoxPit | 5,69 | Intron | ENSCAFG00000031652 | SDC2 | | Syndecan;SDC2 | |
| BoxPit | 4,42 | Coding sequence | ENSCAFG00000025247 | TMEM121B | | Transmembrane protein 121B;TMEM121B | |
| BoxPit | 3,24 | Intron | ENSCAFG00000016100 | - | | Uncharacterized protein;LOC489080 | |
| BoxPit | 3,24 | Intron | ENSCAFG00000023889 | - | | Uncharacterized protein;unassigned | |
| BoxPit | 6,55 | Intron | ENSCAFG00000025023 | - | | Uncharacterized protein;unassigned | |
| BoxPit | 6,55 | Downstream | ENSCAFG00000030880 | - | | Uncharacterized protein;unassigned | |
| BoxPit | 6,84 | Upstream | ENSCAFG00000031827 | - | | Uncharacterized protein;unassigned | |
| BoxPit | 6,84 | Intron | ENSCAFG00000032488 | - | | Uncharacterized protein;unassigned | |
| BoxPit | 6,43 | Non coding | ENSCAFG00000033137 | - | |  | |
| BoxPit | 4,44 | Non coding | ENSCAFG00000035737 | - | |  | |
| BoxPit | 6,84 | Upstream | ENSCAFG00000039645 | - | |  | |
| BoxRot | 6,79 | Downstream | ENSCAFG00000023924 | CD151 | | Tetraspanin;CD151 | |
| BoxWal | -6,50 | Intron | ENSCAFG00000014358 | CASK | | Calcium/calmodulin dependent serine protein kinase;CASK | |
| BoxWal | -6,60 | Intron | ENSCAFG00000014992 | FMO3 | | Dimethylaniline monooxygenase [N-oxide-forming] 3;FMO3 | |
| BoxWal | -6,59 | Intron | ENSCAFG00000018972 | EFCAB5 | | EF-hand calcium binding domain 5;EFCAB5 | |
| BoxWal | -6,63 | Intron | ENSCAFG00000006897 | IL6ST | | Interleukin 6 signal transducer;IL6ST | |
| BoxWal | -6,60 | Intron | ENSCAFG00000014998 | MROH9 | | Maestro heat like repeat family member 9;MROH9 | |
| BoxWal | 7,03 | Upstream | ENSCAFG00000002658 | SLC17A5 | | Solute carrier family 17 member 5;SLC17A5 | |
| BoxWal | -6,52 | Intron | ENSCAFG00000007449 | TRIM23 | | Tripartite motif containing 23;TRIM23 | |
| BoxWal | -6,41 | Intron | ENSCAFG00000031180 | - | | Uncharacterized protein;unassigned | |
| BoxWal | -5,97 | Intron | ENSCAFG00000034656 | - | |  | |
| BoxWal | -6,50 | Intron | ENSCAFG00000035014 | - | |  | |
| BoxWal | -6,18 | Intron | ENSCAFG00000037418 | - | |  | |
| BoxWal | -3,78 | Intron | ENSCAFG00000038613 | - | |  | |
| GerGrea | 8,35 | Upstream | ENSCAFG00000031942 | - | |  | |
| GerLab | -5,84 | Downstream | ENSCAFG00000007870 | DCLK2 | | Doublecortin like kinase 2;DCLK2 | |
| GerLab | -5,96 | Upstream | ENSCAFG00000010688 | - | | Fibroblast growth factor;unassigned | |
| GerLab | 4,32 | Intron | ENSCAFG00000013809 | - | | Uncharacterized protein;unassigned | |
| GerPit | 7,13 | Downstream | ENSCAFG00000023569 | ANKRD60 | | Ankyrin repeat domain 60;ANKRD60 | |
| GerPit | 6,95 | Upstream | ENSCAFG00000008687 | CCT8 | | Chaperonin containing TCP1 subunit 8;CCT8 | |
| GerPit | 5,66 | Downstream | ENSCAFG00000016193 | GDF2 | | Growth differentiation factor 2;GDF2 | |
| GerPit | 5,14 | 3' UTR | ENSCAFG00000011401 | KIRREL1 | | Kirre like nephrin family adhesion molecule 1;KIRREL1 | |
| GerPit | 6,37 | Intron | ENSCAFG00000016038 | LDB3 | | LIM domain binding 3;LDB3 | |
| GerPit | 6,82 | Upstream | ENSCAFG00000008082 | RASSF10 | | Ras association domain family member 10;RASSF10 | |
| GerPit | 7,11 | Downstream | ENSCAFG00000019730 | SLC25A33 | | Solute carrier family 25 member 33;SLC25A33 | |
| GerPit | 7,11 | Upstream | ENSCAFG00000019733 | TMEM201 | | Transmembrane protein 201;TMEM201 | |
| GerPit | 4,96 | Intron | ENSCAFG00000025004 | CYP4A38 | | Uncharacterized protein;CYP4A38 | |
| GerPit | 5,66 | Intron | ENSCAFG00000016100 | - | | Uncharacterized protein;LOC489080 | |
| GerPit | 6,37 | Intron | ENSCAFG00000015966 | WAPL | | WAPL cohesin release factor;WAPL | |
| GerWal | 4,47 | Coding sequence | ENSCAFG00000017991 | C30H15orf39 | | Chromosome 15 open reading frame 39;C30H15orf39 | |
| GerWal | 5,83 | Upstream | ENSCAFG00000014996 | CDK5R2 | | Cyclin-dependent kinase 5 activator;CDK5R2 | |
| GerWal | -3,69 | Intron | ENSCAFG00000016305 | GPHN | | Gephyrin;GPHN | |
| GerWal | -5,90 | Downstream | ENSCAFG00000025596 | KLRK1 | | Killer cell lectin like receptor K1;KLRK1 | |
| GerWal | -4,12 | Intron | ENSCAFG00000011749 | MCCC1 | | Methylcrotonoyl-CoA carboxylase 1;MCCC1 | |
| GerWal | -5,47 | Coding sequence | ENSCAFG00000018274 | MIEF2 | | Mitochondrial elongation factor 2;MIEF2 | |
| GerWal | 6,23 | Coding sequence | ENSCAFG00000003510 | PTPRZ1 | | Protein tyrosine phosphatase, receptor type Z1;PTPRZ1 | |
| GerWal | -2,93 | Intron | ENSCAFG00000014103 | SESTD1 | | SEC14 and spectrin domain containing 1;SESTD1 | |
| GerWal | 6,44 | Intron | ENSCAFG00000031652 | SDC2 | | Syndecan;SDC2 | |
| GerWal | -3,38 | Downstream | ENSCAFG00000017721 | - | | Uncharacterized protein;unassigned | |
| GerWal | -3,12 | Intron | ENSCAFG00000032249 | - | | Uncharacterized protein;unassigned | |
| GerWal | -3,12 | Upstream | ENSCAFG00000027880 | RF00026 | |  | |
| GerWal | -2,08 | Intron | ENSCAFG00000033769 | - | |  | |
| GerWal | -2,40 | Intron | ENSCAFG00000037418 | - | |  | |
| GerWal | 5,83 | Downstream | ENSCAFG00000039386 | - | |  | |
| GreaLab | -6,62 | Intron | ENSCAFG00000010243 | ARL3 | | ADP ribosylation factor like GTPase 3;ARL3 | |
| GreaLab | -6,76 | Downstream | ENSCAFG00000017507 | DOCK6 | | Dedicator of cytokinesis 6;DOCK6 | |
| GreaLab | -6,18 | Upstream | ENSCAFG00000010688 | - | | Fibroblast growth factor;unassigned | |
| GreaLab | -6,76 | Coding sequence | ENSCAFG00000017366 | KANK2 | | KN motif and ankyrin repeat domains 2;KANK2 | |
| GreaLab | -6,94 | Downstream | ENSCAFG00000014309 | RASGRP2 | | RAS guanyl releasing protein 2;RASGRP2 | |
| GreaLab | -7,11 | Intron | ENSCAFG00000002102 | JAK2 | | Tyrosine-protein kinase;JAK2 | |
| GreaLab | 4,01 | Intron | ENSCAFG00000013809 | - | | Uncharacterized protein;unassigned | |
| GreaLab | -6,76 | Upstream | ENSCAFG00000028518 | - | |  | |
| GreaLab | 5,63 | Downstream | ENSCAFG00000036830 | - | |  | |
| GreaLab | -7,11 | Upstream | ENSCAFG00000039255 | - | |  | |
| GreaPit | -6,78 | Downstream | ENSCAFG00000029781 | ABHD16B | | Abhydrolase domain containing 16B;ABHD16B | |
| GreaPit | 6,53 | Upstream | ENSCAFG00000012106 | EMID1 | | EMI domain containing 1;EMID1 | |
| GreaPit | -5,19 | Intron | ENSCAFG00000006255 | NBEA | | Neurobeachin;NBEA | |
| GreaPit | 5,36 | Coding sequence | ENSCAFG00000009036 | PLCB2 | | Phosphoinositide phospholipase C;PLCB2 | |
| GreaPit | 4,97 | Downstream | ENSCAFG00000018282 | ROCK1 | | Rho-associated protein kinase;ROCK1 | |
| GreaPit | -6,78 | Upstream | ENSCAFG00000013072 | TPD52L2 | | Tumor protein D52 like 2;TPD52L2 | |
| GreaPit | 4,97 | Intron | ENSCAFG00000018309 | USP14 | | Ubiquitin specific peptidase 14;USP14 | |
| GreaRot | -6,05 | Intron | ENSCAFG00000010154 | MYH15 | | Myosin heavy chain 15;MYH15 | |
| GreaRot | -5,93 | Intron | ENSCAFG00000015699 | STAMBPL1 | | STAM binding protein like 1;STAMBPL1 | |
| GreaRot | -6,75 | Upstream | ENSCAFG00000010884 | CACNA1S | | Voltage-dependent L-type calcium channel subunit alpha;CACNA1S | |
| GreaRot | 3,50 | Downstream | ENSCAFG00000034455 | - | |  | |
| GreaWal | -5,23 | Intron | ENSCAFG00000011136 | INPP5A | | Inositol polyphosphate-5-phosphatase A;INPP5A | |
| GreaWal | -5,45 | Intron | ENSCAFG00000012720 | NTSR1 | | Neurotensin receptor 1;NTSR1 | |
| GreaWal | 6,87 | Intron | ENSCAFG00000018073 | PSTPIP1 | | Proline-serine-threonine phosphatase interacting protein 1;PSTPIP1 | |
| GreaWal | -5,05 | Upstream | ENSCAFG00000012704 | RBBP8NL | | RBBP8 N-terminal like;RBBP8NL | |
| GreaWal | -6,55 | Intron | ENSCAFG00000018462 | RNF135 | | Ring finger protein 135;RNF135 | |
| GreaWal | -5,05 | Intron | ENSCAFG00000012668 | - | | Uncharacterized protein;unassigned | |
| GreaWal | -6,20 | Intron | ENSCAFG00000029547 | - | | Uncharacterized protein;unassigned | |
| GreaWal | -5,23 | Intron | ENSCAFG00000031153 | - | | Uncharacterized protein;unassigned | |
| GreaWal | -6,20 | Intron | ENSCAFG00000031797 | - | | Uncharacterized protein;unassigned | |
| GreaWal | -5,44 | Coding sequence | ENSCAFG00000013099 | UCKL1 | | Uridine-cytidine kinase;UCKL1 | |
| LabPit | 7,05 | Intron | ENSCAFG00000010243 | ARL3 | | ADP ribosylation factor like GTPase 3;ARL3 | |
| LabPit | 6,23 | Upstream | ENSCAFG00000019881 | CYBA | | Cytochrome b-245 light chain;CYBA | |
| LabPit | 7,20 | Downstream | ENSCAFG00000017507 | DOCK6 | | Dedicator of cytokinesis 6;DOCK6 | |
| LabPit | 6,23 | 3' UTR | ENSCAFG00000019878 | MVD | | Diphosphomevalonate decarboxylase;MVD | |
| LabPit | 6,66 | Upstream | ENSCAFG00000010688 | - | | Fibroblast growth factor;unassigned | |
| LabPit | 4,31 | Downstream | ENSCAFG00000016130 | ILVBL | | IlvB acetolactate synthase like;ILVBL | |
| LabPit | 7,20 | Intron | ENSCAFG00000017366 | KANK2 | | KN motif and ankyrin repeat domains 2;KANK2 | |
| LabPit | 5,82 | Intron | ENSCAFG00000005114 | MYRIP | | Myosin VIIA and Rab interacting protein;MYRIP | |
| LabPit | 6,52 | Intron | ENSCAFG00000012602 | OSBPL2 | | Oxysterol-binding protein;OSBPL2 | |
| LabPit | 6,03 | Downstream | ENSCAFG00000000699 | PANX2 | | Pannexin;PANX2 | |
| LabPit | 7,84 | Intron | ENSCAFG00000003228 | PXDN | | Peroxidasin;PXDN | |
| LabPit | 6,71 | Downstream | ENSCAFG00000014309 | RASGRP2 | | RAS guanyl releasing protein 2;RASGRP2 | |
| LabPit | 4,31 | Intron | ENSCAFG00000016147 | SYDE1 | | Synapse defective Rho GTPase homolog 1;SYDE1 | |
| LabPit | 6,22 | Intron | ENSCAFG00000018445 | TEDC1 | | Tubulin epsilon and delta complex 1;TEDC1 | |
| LabPit | 7,56 | 5' UTR | ENSCAFG00000002102 | JAK2 | | Tyrosine-protein kinase;JAK2 | |
| LabPit | 6,52 | Coding sequence | ENSCAFG00000012668 | - | | Uncharacterized protein;unassigned | |
| LabPit | 6,23 | Intron | ENSCAFG00000029455 | - | | Uncharacterized protein;unassigned | |
| LabPit | 6,24 | Coding sequence | ENSCAFG00000030503 | - | | Uncharacterized protein;unassigned | |
| LabPit | 6,22 | Downstream | ENSCAFG00000031417 | - | | Uncharacterized protein;unassigned | |
| LabPit | 7,20 | Upstream | ENSCAFG00000028518 | - | |  | |
| LabPit | 6,24 | Intron | ENSCAFG00000032882 | - | |  | |
| LabPit | -6,12 | Downstream | ENSCAFG00000036830 | - | |  | |
| LabPit | 2,71 | Non coding | ENSCAFG00000037156 | - | |  | |
| LabPit | 7,18 | Intron | ENSCAFG00000039151 | - | |  | |
| LabPit | 7,56 | Upstream | ENSCAFG00000039255 | - | |  | |
| LabPit | -3,43 | Intron | ENSCAFG00000040354 | - | |  | |
| LabRot | 6,41 | Downstream | ENSCAFG00000016701 | IDUA | | Alpha-L-iduronidase;IDUA | |
| LabRot | -4,01 | Intron | ENSCAFG00000013809 | - | | Uncharacterized protein;unassigned | |
| LabWal | 3,55 | Downstream | ENSCAFG00000007870 | DCLK2 | | Doublecortin like kinase 2;DCLK2 | |
| LabWal | -5,91 | Downstream | ENSCAFG00000025596 | KLRK1 | | Killer cell lectin like receptor K1;KLRK1 | |
| LabWal | -5,85 | Intron | ENSCAFG00000004279 | MYO3A | | Myosin IIIA;MYO3A | |
| LabWal | -5,62 | Intron | ENSCAFG00000010498 | KCNH7 | | Potassium voltage-gated channel subfamily H member 7;KCNH7 | |
| LabWal | 5,86 | Coding sequence | ENSCAFG00000003510 | PTPRZ1 | | Protein tyrosine phosphatase, receptor type Z1;PTPRZ1 | |
| LabWal | 6,73 | Coding sequence | ENSCAFG00000019552 | ARHGAP45 | | Rho GTPase activating protein 45;ARHGAP45 | |
| LabWal | 3,55 | Coding sequence | ENSCAFG00000000013 | SALL3 | | Spalt like transcription factor 3;SALL3 | |
| LabWal | -6,15 | Downstream | ENSCAFG00000017721 | - | | Uncharacterized protein;unassigned | |
| LabWal | -5,71 | Downstream | ENSCAFG00000022238 | RF00026 | |  | |
| PitRot | -5,67 | Intron | ENSCAFG00000015182 | GLB1L | | Galactosidase beta 1 like;GLB1L | |
| PitRot | -7,20 | 3' UTR | ENSCAFG00000016295 | GIPC1 | | GIPC PDZ domain containing family member 1;GIPC1 | |
| PitRot | -5,46 | Intron | ENSCAFG00000024839 | KIF21B | | Kinesin family member 21B;KIF21B | |
| PitRot | -4,78 | Upstream | ENSCAFG00000016473 | NSD1 | | Nuclear receptor binding SET domain protein 1;NSD1 | |
| PitRot | -6,51 | Intron | ENSCAFG00000019602 | PLEKHG5 | | Pleckstrin homology and RhoGEF domain containing G5;PLEKHG5 | |
| PitRot | -7,20 | Upstream | ENSCAFG00000016300 | PTGER1 | | Prostaglandin E2 receptor EP1 subtype;PTGER1 | |
| PitRot | -7,20 | Downstream | ENSCAFG00000016316 | PKN1 | | Protein kinase N1;PKN1 | |
| PitRot | -5,67 | Upstream | ENSCAFG00000015200 | STK16 | | Serine/threonine kinase 16;STK16 | |
| PitRot | -6,81 | Downstream | ENSCAFG00000019730 | SLC25A33 | | Solute carrier family 25 member 33;SLC25A33 | |
| PitRot | -6,81 | Upstream | ENSCAFG00000019733 | TMEM201 | | Transmembrane protein 201;TMEM201 | |
| PitRot | -7,20 | Downstream | ENSCAFG00000029373 | - | | Uncharacterized protein;unassigned | |
| PitRot | -5,89 | Coding sequence | ENSCAFG00000020243 | ZFHX3 | | Zinc finger homeobox 3;ZFHX3 | |
| PitWal | -2,73 | Coding sequence | ENSCAFG00000018274 | MIEF2 | | Mitochondrial elongation factor 2;MIEF2 | |
| PitWal | -6,76 | Coding sequence | ENSCAFG00000018462 | RNF135 | | Ring finger protein 135;RNF135 | |
| PitWal | -5,10 | Coding sequence | ENSCAFG00000000652 | TULP4 | | Tubby like protein 4;TULP4 | |
| PitWal | 6,40 | Downstream | ENSCAFG00000024680 | - | | Uncharacterized protein;unassigned | |
| PitWal | -6,38 | Intron | ENSCAFG00000029547 | - | | Uncharacterized protein;unassigned | |
| PitWal | -6,38 | Intron | ENSCAFG00000031797 | - | | Uncharacterized protein;unassigned | |
| PitWal | -5,26 | Coding sequence | ENSCAFG00000013099 | UCKL1 | | Uridine-cytidine kinase;UCKL1 | |
| PitWal | 6,40 | Downstream | ENSCAFG00000022702 | RF00026 | |  | |
| RotWal | 6,76 | Downstream | ENSCAFG00000016101 | GPRIN2 | | G protein regulated inducer of neurite outgrowth 2;GPRIN2 | |
| RotWal | 5,65 | Downstream | ENSCAFG00000032024 | GPR162 | | G protein-coupled receptor 162;GPR162 | |
| RotWal | 7,28 | 3' UTR | ENSCAFG00000016295 | GIPC1 | | GIPC PDZ domain containing family member 1;GIPC1 | |
| RotWal | -3,06 | Downstream | ENSCAFG00000025596 | KLRK1 | | Killer cell lectin like receptor K1;KLRK1 | |
| RotWal | 6,42 | Intron | ENSCAFG00000010154 | MYH15 | | Myosin heavy chain 15;MYH15 | |
| RotWal | 6,18 | Downstream | ENSCAFG00000018404 | NUDT14 | | Nudix hydrolase 14;NUDT14 | |
| RotWal | 6,57 | Intron | ENSCAFG00000019602 | PLEKHG5 | | Pleckstrin homology and RhoGEF domain containing G5;PLEKHG5 | |
| RotWal | 5,65 | Coding sequence | ENSCAFG00000031318 | P3H3 | | Prolyl 3-hydroxylase 3;P3H3 | |
| RotWal | 7,28 | Upstream | ENSCAFG00000016300 | PTGER1 | | Prostaglandin E2 receptor EP1 subtype;PTGER1 | |
| RotWal | 7,28 | Downstream | ENSCAFG00000016316 | PKN1 | | Protein kinase N1;PKN1 | |
| RotWal | 6,76 | Intron | ENSCAFG00000016100 | - | | Uncharacterized protein;LOC489080 | |
| RotWal | 7,28 | Downstream | ENSCAFG00000029373 | - | | Uncharacterized protein;unassigned | |
| RotWal | 6,05 | Downstream | ENSCAFG00000031597 | - | | Uncharacterized protein;unassigned | |
| RotWal | 6,05 | Intron | ENSCAFG00000015484 | WSCD1 | | WSC domain containing 1;WSCD1 | |
| RotWal | 6,18 | Downstream | ENSCAFG00000038945 | - | |  | |
|  | | | | |  | |  |
|  | | | | |  | |  |
