# Supplementary material for: DNA methylation in canine brains is related to domestication and dog-breed formation
Source: PLoS One. 2020 Oct 29;15(10):e0240787. doi: 10.1371/journal.pone.0240787 (PMC7595415; doi:10.1371/journal.pone.0240787)
Supplement: S4 Table — (DOCX) [file pone.0240787.s004.docx]

| **S4 Table.** High level gene ontology categories from ShinyGO v0.50 for genes with DMRs from the breed comparisons. | |
| --- | --- |
| **High level GO category** | **Genes** |
| Activation of immune response | ENSCAFG00000018608 ENSCAFG00000019881 ENSCAFG00000025596 |
| Anatomical structure development | ENSCAFG00000000013 ENSCAFG00000002102 ENSCAFG00000007601 ENSCAFG00000007870 ENSCAFG00000016193 ENSCAFG00000016292 ENSCAFG00000024680 ENSCAFG00000030240 ENSCAFG00000030503 ENSCAFG00000031652 ENSCAFG00000000163 ENSCAFG00000003354 ENSCAFG00000003510 ENSCAFG00000005811 ENSCAFG00000006897 ENSCAFG00000010243 ENSCAFG00000014996 ENSCAFG00000015753 ENSCAFG00000016016 ENSCAFG00000016038 ENSCAFG00000016147 ENSCAFG00000016316 ENSCAFG00000018119 ENSCAFG00000018274 ENSCAFG00000018282 ENSCAFG00000019927 ENSCAFG00000023431 ENSCAFG00000023924 ENSCAFG00000031536 ENSCAFG00000031660 ENSCAFG00000032121 |
| Anatomical structure formation involved in morphogenesis | ENSCAFG00000030240 ENSCAFG00000014996 ENSCAFG00000016038 ENSCAFG00000016193 ENSCAFG00000016316 ENSCAFG00000018119 ENSCAFG00000018282 ENSCAFG00000023431 |
| Anatomical structure morphogenesis | ENSCAFG00000030240 ENSCAFG00000031652 ENSCAFG00000000013 ENSCAFG00000000163 ENSCAFG00000003510 ENSCAFG00000005811 ENSCAFG00000014996 ENSCAFG00000016038 ENSCAFG00000016193 ENSCAFG00000016316 ENSCAFG00000018119 ENSCAFG00000018274 ENSCAFG00000018282 ENSCAFG00000019927 ENSCAFG00000023431 ENSCAFG00000023924 ENSCAFG00000031660 |
| Behavior | ENSCAFG00000003510 ENSCAFG00000003882 ENSCAFG00000012720 ENSCAFG00000016292 ENSCAFG00000023431 |
| Biological adhesion | ENSCAFG00000002102 ENSCAFG00000016292 ENSCAFG00000003354 ENSCAFG00000006532 ENSCAFG00000006897 ENSCAFG00000011401 ENSCAFG00000015753 ENSCAFG00000018282 |
| Biosynthetic process | ENSCAFG00000000013 ENSCAFG00000007601 ENSCAFG00000013099 ENSCAFG00000016193 ENSCAFG00000016305 ENSCAFG00000019878 ENSCAFG00000024680 ENSCAFG00000031827 ENSCAFG00000032488 ENSCAFG00000000163 ENSCAFG00000003354 ENSCAFG00000007614 ENSCAFG00000007662 ENSCAFG00000008687 ENSCAFG00000014350 ENSCAFG00000014358 ENSCAFG00000015966 ENSCAFG00000016016 ENSCAFG00000016316 ENSCAFG00000016473 ENSCAFG00000016708 ENSCAFG00000017366 ENSCAFG00000018119 ENSCAFG00000019730 ENSCAFG00000019881 ENSCAFG00000020243 ENSCAFG00000025596 ENSCAFG00000031318 |
| Catabolic process | ENSCAFG00000003228 ENSCAFG00000003882 ENSCAFG00000009036 ENSCAFG00000016016 ENSCAFG00000016295 ENSCAFG00000016701 ENSCAFG00000018282 ENSCAFG00000018309 ENSCAFG00000019382 ENSCAFG00000032704 |
| Cell adhesion | ENSCAFG00000002102 ENSCAFG00000016292 ENSCAFG00000003354 ENSCAFG00000006532 ENSCAFG00000006897 ENSCAFG00000011401 ENSCAFG00000018282 |
| Cell cycle process | ENSCAFG00000015966 ENSCAFG00000007614 ENSCAFG00000010243 ENSCAFG00000016295 ENSCAFG00000017366 ENSCAFG00000019730 |
| Cell growth | ENSCAFG00000016193 ENSCAFG00000019730 |
| Cell motility | ENSCAFG00000002102 ENSCAFG00000031652 ENSCAFG00000014996 ENSCAFG00000016147 ENSCAFG00000016193 ENSCAFG00000016295 ENSCAFG00000016316 ENSCAFG00000018282 ENSCAFG00000019602 ENSCAFG00000019733 ENSCAFG00000023924 ENSCAFG00000025596 ENSCAFG00000031536 |
| Cell proliferation | ENSCAFG00000002102 ENSCAFG00000000163 ENSCAFG00000003510 ENSCAFG00000006897 ENSCAFG00000015966 ENSCAFG00000016193 ENSCAFG00000016316 ENSCAFG00000017366 ENSCAFG00000019730 ENSCAFG00000019878 ENSCAFG00000023924 ENSCAFG00000031318 |
| Cellular component biogenesis | ENSCAFG00000016292 ENSCAFG00000008687 ENSCAFG00000010243 ENSCAFG00000011401 ENSCAFG00000016038 ENSCAFG00000016708 ENSCAFG00000018274 ENSCAFG00000018282 ENSCAFG00000019730 ENSCAFG00000019881 ENSCAFG00000023431 ENSCAFG00000031536 ENSCAFG00000032704 |
| Cellular component organization | ENSCAFG00000007870 ENSCAFG00000015966 ENSCAFG00000016100 ENSCAFG00000016292 ENSCAFG00000016305 ENSCAFG00000031652 ENSCAFG00000002102 ENSCAFG00000003228 ENSCAFG00000003510 ENSCAFG00000005811 ENSCAFG00000007614 ENSCAFG00000008687 ENSCAFG00000010243 ENSCAFG00000011401 ENSCAFG00000016038 ENSCAFG00000016147 ENSCAFG00000016193 ENSCAFG00000016316 ENSCAFG00000016473 ENSCAFG00000016708 ENSCAFG00000018274 ENSCAFG00000018282 ENSCAFG00000019730 ENSCAFG00000019881 ENSCAFG00000019927 ENSCAFG00000023431 ENSCAFG00000023924 ENSCAFG00000031536 ENSCAFG00000031660 ENSCAFG00000032704 |
| Cellular component organization or biogenesis | ENSCAFG00000007870 ENSCAFG00000015966 ENSCAFG00000016100 ENSCAFG00000016292 ENSCAFG00000016305 ENSCAFG00000031652 ENSCAFG00000002102 ENSCAFG00000003228 ENSCAFG00000003510 ENSCAFG00000005811 ENSCAFG00000007614 ENSCAFG00000008687 ENSCAFG00000010243 ENSCAFG00000011401 ENSCAFG00000016038 ENSCAFG00000016147 ENSCAFG00000016193 ENSCAFG00000016316 ENSCAFG00000016473 ENSCAFG00000016708 ENSCAFG00000018274 ENSCAFG00000018282 ENSCAFG00000019730 ENSCAFG00000019881 ENSCAFG00000019927 ENSCAFG00000023431 ENSCAFG00000023924 ENSCAFG00000031536 ENSCAFG00000031660 ENSCAFG00000032704 |
| Cellular localization | ENSCAFG00000000652 ENSCAFG00000005114 ENSCAFG00000015966 ENSCAFG00000016100 ENSCAFG00000016292 ENSCAFG00000016305 ENSCAFG00000017721 ENSCAFG00000007614 ENSCAFG00000007870 ENSCAFG00000008687 ENSCAFG00000010243 ENSCAFG00000016295 ENSCAFG00000018274 ENSCAFG00000018282 ENSCAFG00000019733 ENSCAFG00000019881 ENSCAFG00000032520 |
| Developmental process | ENSCAFG00000000013 ENSCAFG00000002102 ENSCAFG00000007601 ENSCAFG00000007870 ENSCAFG00000016193 ENSCAFG00000016292 ENSCAFG00000024680 ENSCAFG00000030240 ENSCAFG00000030503 ENSCAFG00000031652 ENSCAFG00000000163 ENSCAFG00000003354 ENSCAFG00000003510 ENSCAFG00000005811 ENSCAFG00000006897 ENSCAFG00000010243 ENSCAFG00000014996 ENSCAFG00000015753 ENSCAFG00000016016 ENSCAFG00000016038 ENSCAFG00000016147 ENSCAFG00000016316 ENSCAFG00000018119 ENSCAFG00000018274 ENSCAFG00000018282 ENSCAFG00000019927 ENSCAFG00000023431 ENSCAFG00000023924 ENSCAFG00000031536 ENSCAFG00000031660 ENSCAFG00000032121 |
| Establishment of localization | ENSCAFG00000000699 ENSCAFG00000002658 ENSCAFG00000005114 ENSCAFG00000010665 ENSCAFG00000016100 ENSCAFG00000016292 ENSCAFG00000017721 ENSCAFG00000019730 ENSCAFG00000031660 ENSCAFG00000001907 ENSCAFG00000007662 ENSCAFG00000008687 ENSCAFG00000010243 ENSCAFG00000010498 ENSCAFG00000010884 ENSCAFG00000012602 ENSCAFG00000014103 ENSCAFG00000014358 ENSCAFG00000014996 ENSCAFG00000016295 ENSCAFG00000018274 ENSCAFG00000018282 ENSCAFG00000019374 ENSCAFG00000019733 ENSCAFG00000019881 ENSCAFG00000023924 ENSCAFG00000029188 ENSCAFG00000032520 ENSCAFG00000032704 |
| Growth | ENSCAFG00000016193 ENSCAFG00000019730 |
| Hormone metabolic process | ENSCAFG00000001907 ENSCAFG00000003882 ENSCAFG00000016708 |
| Immune effector process | ENSCAFG00000000163 ENSCAFG00000016316 ENSCAFG00000018608 ENSCAFG00000024030 ENSCAFG00000025596 |
| Immune response | ENSCAFG00000002102 ENSCAFG00000032249 ENSCAFG00000000163 ENSCAFG00000015753 ENSCAFG00000016316 ENSCAFG00000018462 ENSCAFG00000018608 ENSCAFG00000019881 ENSCAFG00000024030 ENSCAFG00000025596 |
| Immune system development | ENSCAFG00000002102 ENSCAFG00000000163 ENSCAFG00000003510 ENSCAFG00000016316 |
| Immune system process | ENSCAFG00000002102 ENSCAFG00000024030 ENSCAFG00000032249 ENSCAFG00000000163 ENSCAFG00000003510 ENSCAFG00000006897 ENSCAFG00000015753 ENSCAFG00000016316 ENSCAFG00000018282 ENSCAFG00000018462 ENSCAFG00000018608 ENSCAFG00000019881 ENSCAFG00000023924 ENSCAFG00000025596 ENSCAFG00000031660 |
| Leukocyte activation | ENSCAFG00000000163 ENSCAFG00000006897 ENSCAFG00000016316 ENSCAFG00000023924 ENSCAFG00000024030 ENSCAFG00000025596 |
| Leukocyte homeostasis | ENSCAFG00000016316 ENSCAFG00000031660 |
| Leukocyte migration | ENSCAFG00000018282 ENSCAFG00000025596 |
| Localization | ENSCAFG00000000652 ENSCAFG00000000699 ENSCAFG00000002102 ENSCAFG00000002658 ENSCAFG00000005114 ENSCAFG00000010665 ENSCAFG00000015966 ENSCAFG00000016100 ENSCAFG00000016292 ENSCAFG00000016305 ENSCAFG00000017721 ENSCAFG00000019730 ENSCAFG00000031652 ENSCAFG00000031660 ENSCAFG00000001907 ENSCAFG00000006255 ENSCAFG00000007614 ENSCAFG00000007662 ENSCAFG00000007870 ENSCAFG00000008687 ENSCAFG00000010243 ENSCAFG00000010498 ENSCAFG00000010884 ENSCAFG00000012602 ENSCAFG00000014103 ENSCAFG00000014358 ENSCAFG00000014996 ENSCAFG00000016147 ENSCAFG00000016193 ENSCAFG00000016295 ENSCAFG00000016316 ENSCAFG00000018274 ENSCAFG00000018282 ENSCAFG00000019374 ENSCAFG00000019602 ENSCAFG00000019733 ENSCAFG00000019881 ENSCAFG00000023924 ENSCAFG00000025596 ENSCAFG00000029188 ENSCAFG00000031536 ENSCAFG00000032520 ENSCAFG00000032704 |
| Localization of cell | ENSCAFG00000002102 ENSCAFG00000031652 ENSCAFG00000014996 ENSCAFG00000016147 ENSCAFG00000016193 ENSCAFG00000016295 ENSCAFG00000016316 ENSCAFG00000018282 ENSCAFG00000019602 ENSCAFG00000019733 ENSCAFG00000023924 ENSCAFG00000025596 ENSCAFG00000031536 |
| Locomotion | ENSCAFG00000002102 ENSCAFG00000031652 ENSCAFG00000014996 ENSCAFG00000016147 ENSCAFG00000016193 ENSCAFG00000016295 ENSCAFG00000016316 ENSCAFG00000018282 ENSCAFG00000018309 ENSCAFG00000019602 ENSCAFG00000019733 ENSCAFG00000023924 ENSCAFG00000025596 ENSCAFG00000031536 |
| Locomotory behavior | ENSCAFG00000012720 ENSCAFG00000016292 |
| Macromolecule localization | ENSCAFG00000000652 ENSCAFG00000015966 ENSCAFG00000016305 ENSCAFG00000017721 ENSCAFG00000005114 ENSCAFG00000006255 ENSCAFG00000007614 ENSCAFG00000007662 ENSCAFG00000007870 ENSCAFG00000008687 ENSCAFG00000012602 ENSCAFG00000016292 ENSCAFG00000016295 ENSCAFG00000018274 ENSCAFG00000018282 ENSCAFG00000029188 ENSCAFG00000032520 |
| Meiotic cell cycle process | ENSCAFG00000015966 ENSCAFG00000007614 |
| Multicellular organism reproduction | ENSCAFG00000000612 ENSCAFG00000003882 |
| Multicellular organismal reproductive process | ENSCAFG00000000612 ENSCAFG00000003882 |
| Multi-multicellular organism process | ENSCAFG00000001907 ENSCAFG00000003882 ENSCAFG00000016147 |
| Multi-organism process | ENSCAFG00000000612 ENSCAFG00000001907 ENSCAFG00000003882 ENSCAFG00000008687 ENSCAFG00000015753 ENSCAFG00000016147 ENSCAFG00000016300 ENSCAFG00000019881 ENSCAFG00000025596 |
| Multi-organism reproductive process | ENSCAFG00000000612 ENSCAFG00000001907 ENSCAFG00000003882 ENSCAFG00000008687 ENSCAFG00000016147 |
| Negative regulation of biological process | ENSCAFG00000024680 ENSCAFG00000000013 ENSCAFG00000000163 ENSCAFG00000003354 ENSCAFG00000007601 ENSCAFG00000007614 ENSCAFG00000007870 ENSCAFG00000011401 ENSCAFG00000012720 ENSCAFG00000014103 ENSCAFG00000015966 ENSCAFG00000016016 ENSCAFG00000016193 ENSCAFG00000016295 ENSCAFG00000016316 ENSCAFG00000016708 ENSCAFG00000017366 ENSCAFG00000018282 ENSCAFG00000018309 ENSCAFG00000019382 ENSCAFG00000019730 ENSCAFG00000019881 ENSCAFG00000020243 ENSCAFG00000023431 ENSCAFG00000025596 ENSCAFG00000030240 ENSCAFG00000031318 ENSCAFG00000031536 ENSCAFG00000032704 |
| Neurotransmitter secretion | ENSCAFG00000016100 ENSCAFG00000032520 |
| Positive regulation of biological process | ENSCAFG00000016193 ENSCAFG00000000163 ENSCAFG00000002102 ENSCAFG00000003354 ENSCAFG00000006897 ENSCAFG00000007662 ENSCAFG00000008687 ENSCAFG00000011401 ENSCAFG00000014350 ENSCAFG00000014358 ENSCAFG00000014996 ENSCAFG00000015966 ENSCAFG00000016147 ENSCAFG00000016292 ENSCAFG00000016295 ENSCAFG00000016473 ENSCAFG00000016708 ENSCAFG00000018274 ENSCAFG00000018282 ENSCAFG00000018462 ENSCAFG00000018608 ENSCAFG00000019382 ENSCAFG00000019730 ENSCAFG00000019878 ENSCAFG00000019881 ENSCAFG00000020243 ENSCAFG00000023431 ENSCAFG00000023924 ENSCAFG00000025596 |
| Presynaptic process involved in chemical synaptic transmission | ENSCAFG00000016100 ENSCAFG00000032520 |
| Production of molecular mediator of immune response | ENSCAFG00000032249 ENSCAFG00000000163 ENSCAFG00000016316 |
| Regulation of biological quality | ENSCAFG00000002102 ENSCAFG00000010498 ENSCAFG00000010884 ENSCAFG00000016100 ENSCAFG00000001907 ENSCAFG00000003882 ENSCAFG00000007614 ENSCAFG00000008687 ENSCAFG00000011401 ENSCAFG00000015753 ENSCAFG00000016016 ENSCAFG00000016292 ENSCAFG00000016295 ENSCAFG00000016316 ENSCAFG00000016708 ENSCAFG00000019730 ENSCAFG00000019881 ENSCAFG00000023431 ENSCAFG00000025596 ENSCAFG00000031660 ENSCAFG00000032520 ENSCAFG00000032704 |
| Regulation of cell adhesion | ENSCAFG00000002102 ENSCAFG00000003354 ENSCAFG00000006897 ENSCAFG00000018282 |
| Regulation of cellular component biogenesis | ENSCAFG00000011401 ENSCAFG00000018274 ENSCAFG00000018282 ENSCAFG00000023431 ENSCAFG00000031536 |
| Regulation of developmental process | ENSCAFG00000024680 ENSCAFG00000000163 ENSCAFG00000003354 ENSCAFG00000003510 ENSCAFG00000005811 ENSCAFG00000006897 ENSCAFG00000007601 ENSCAFG00000016147 ENSCAFG00000016193 ENSCAFG00000016316 ENSCAFG00000018274 ENSCAFG00000018282 ENSCAFG00000023431 ENSCAFG00000031536 ENSCAFG00000032121 |
| Regulation of growth | ENSCAFG00000016193 ENSCAFG00000019730 |
| Regulation of immune system process | ENSCAFG00000000163 ENSCAFG00000006897 ENSCAFG00000016316 ENSCAFG00000018462 ENSCAFG00000018608 ENSCAFG00000019881 ENSCAFG00000025596 |
| Regulation of localization | ENSCAFG00000015966 ENSCAFG00000016100 ENSCAFG00000001907 ENSCAFG00000007614 ENSCAFG00000007870 ENSCAFG00000008687 ENSCAFG00000010498 ENSCAFG00000010884 ENSCAFG00000014103 ENSCAFG00000014358 ENSCAFG00000014996 ENSCAFG00000016147 ENSCAFG00000016193 ENSCAFG00000016292 ENSCAFG00000016316 ENSCAFG00000018274 ENSCAFG00000018282 ENSCAFG00000019881 ENSCAFG00000023924 ENSCAFG00000025596 ENSCAFG00000031536 |
| Regulation of locomotion | ENSCAFG00000016147 ENSCAFG00000016193 ENSCAFG00000016316 ENSCAFG00000018309 ENSCAFG00000023924 ENSCAFG00000025596 ENSCAFG00000031536 |
| Regulation of metabolic process | ENSCAFG00000000013 ENSCAFG00000016193 ENSCAFG00000024680 ENSCAFG00000031827 ENSCAFG00000032488 ENSCAFG00000000163 ENSCAFG00000003354 ENSCAFG00000006897 ENSCAFG00000007601 ENSCAFG00000007614 ENSCAFG00000007662 ENSCAFG00000008687 ENSCAFG00000011401 ENSCAFG00000014350 ENSCAFG00000014358 ENSCAFG00000014996 ENSCAFG00000015966 ENSCAFG00000016016 ENSCAFG00000016295 ENSCAFG00000016316 ENSCAFG00000016473 ENSCAFG00000016708 ENSCAFG00000017366 ENSCAFG00000018119 ENSCAFG00000018282 ENSCAFG00000018309 ENSCAFG00000019382 ENSCAFG00000019730 ENSCAFG00000019881 ENSCAFG00000020243 ENSCAFG00000025596 ENSCAFG00000030240 ENSCAFG00000031536 ENSCAFG00000032121 ENSCAFG00000032704 |
| Regulation of molecular function | ENSCAFG00000015966 ENSCAFG00000016147 ENSCAFG00000000612 ENSCAFG00000007614 ENSCAFG00000007662 ENSCAFG00000010688 ENSCAFG00000014309 ENSCAFG00000014996 ENSCAFG00000015753 ENSCAFG00000016193 ENSCAFG00000016292 ENSCAFG00000016316 ENSCAFG00000016473 ENSCAFG00000016708 ENSCAFG00000017507 ENSCAFG00000018282 ENSCAFG00000018309 ENSCAFG00000019552 ENSCAFG00000019602 ENSCAFG00000023431 ENSCAFG00000025596 ENSCAFG00000030240 ENSCAFG00000030366 |
| Regulation of multicellular organismal process | ENSCAFG00000000163 ENSCAFG00000001907 ENSCAFG00000003354 ENSCAFG00000003510 ENSCAFG00000006897 ENSCAFG00000007601 ENSCAFG00000016147 ENSCAFG00000016193 ENSCAFG00000016292 ENSCAFG00000018282 ENSCAFG00000018462 ENSCAFG00000019881 ENSCAFG00000023431 ENSCAFG00000025596 ENSCAFG00000031536 |
| Regulation of multi-organism process | ENSCAFG00000015753 ENSCAFG00000016147 ENSCAFG00000019881 |
| Regulation of response to stimulus | ENSCAFG00000016193 ENSCAFG00000024680 ENSCAFG00000000013 ENSCAFG00000000163 ENSCAFG00000002102 ENSCAFG00000003354 ENSCAFG00000006897 ENSCAFG00000007601 ENSCAFG00000010688 ENSCAFG00000016292 ENSCAFG00000016295 ENSCAFG00000016316 ENSCAFG00000016708 ENSCAFG00000017366 ENSCAFG00000018309 ENSCAFG00000018462 ENSCAFG00000018608 ENSCAFG00000019382 ENSCAFG00000019602 ENSCAFG00000019881 ENSCAFG00000025596 ENSCAFG00000030240 ENSCAFG00000031536 |
| Regulation of signaling | ENSCAFG00000016193 ENSCAFG00000016292 ENSCAFG00000024680 ENSCAFG00000000013 ENSCAFG00000003354 ENSCAFG00000006897 ENSCAFG00000007601 ENSCAFG00000010688 ENSCAFG00000016295 ENSCAFG00000016708 ENSCAFG00000017366 ENSCAFG00000019602 ENSCAFG00000019881 ENSCAFG00000030240 ENSCAFG00000031536 |
| Reproduction | ENSCAFG00000015966 ENSCAFG00000000612 ENSCAFG00000001907 ENSCAFG00000003882 ENSCAFG00000007614 ENSCAFG00000008687 ENSCAFG00000016147 |
| Reproductive process | ENSCAFG00000015966 ENSCAFG00000000612 ENSCAFG00000001907 ENSCAFG00000003882 ENSCAFG00000007614 ENSCAFG00000008687 ENSCAFG00000016147 |
| Response to abiotic stimulus | ENSCAFG00000016016 ENSCAFG00000016316 |
| Response to biotic stimulus | ENSCAFG00000015753 ENSCAFG00000016300 ENSCAFG00000019881 ENSCAFG00000025596 |
| Response to chemical | ENSCAFG00000002102 ENSCAFG00000015200 ENSCAFG00000016193 ENSCAFG00000024680 ENSCAFG00000000163 ENSCAFG00000003228 ENSCAFG00000003354 ENSCAFG00000006897 ENSCAFG00000007662 ENSCAFG00000010688 ENSCAFG00000014309 ENSCAFG00000015966 ENSCAFG00000016295 ENSCAFG00000016300 ENSCAFG00000016305 ENSCAFG00000016708 ENSCAFG00000017366 ENSCAFG00000018309 ENSCAFG00000019382 ENSCAFG00000019602 ENSCAFG00000019730 ENSCAFG00000020243 ENSCAFG00000025596 ENSCAFG00000031536 ENSCAFG00000032520 |
| Response to endogenous stimulus | ENSCAFG00000015200 ENSCAFG00000016193 ENSCAFG00000024680 ENSCAFG00000002102 ENSCAFG00000003354 ENSCAFG00000007662 ENSCAFG00000010688 ENSCAFG00000016295 ENSCAFG00000017366 ENSCAFG00000019730 ENSCAFG00000020243 ENSCAFG00000031536 |
| Response to external stimulus | ENSCAFG00000002102 ENSCAFG00000015753 ENSCAFG00000016300 ENSCAFG00000016708 ENSCAFG00000017366 ENSCAFG00000018309 ENSCAFG00000019602 ENSCAFG00000019881 ENSCAFG00000025596 |
| Response to other organism | ENSCAFG00000015753 ENSCAFG00000016300 ENSCAFG00000019881 ENSCAFG00000025596 |
| Response to stress | ENSCAFG00000002102 ENSCAFG00000000699 ENSCAFG00000003228 ENSCAFG00000015753 ENSCAFG00000016016 ENSCAFG00000016316 ENSCAFG00000018309 ENSCAFG00000018462 ENSCAFG00000019382 ENSCAFG00000019881 ENSCAFG00000023924 ENSCAFG00000025596 |
| Sexual reproduction | ENSCAFG00000000612 ENSCAFG00000003882 ENSCAFG00000008687 |
| Signaling | ENSCAFG00000000013 ENSCAFG00000000699 ENSCAFG00000002102 ENSCAFG00000006686 ENSCAFG00000007601 ENSCAFG00000007870 ENSCAFG00000016100 ENSCAFG00000016193 ENSCAFG00000016292 ENSCAFG00000016316 ENSCAFG00000023924 ENSCAFG00000024578 ENSCAFG00000024680 ENSCAFG00000000163 ENSCAFG00000003354 ENSCAFG00000006897 ENSCAFG00000007614 ENSCAFG00000007662 ENSCAFG00000008082 ENSCAFG00000009036 ENSCAFG00000010243 ENSCAFG00000010688 ENSCAFG00000012720 ENSCAFG00000013632 ENSCAFG00000014309 ENSCAFG00000016147 ENSCAFG00000016295 ENSCAFG00000016300 ENSCAFG00000016708 ENSCAFG00000017366 ENSCAFG00000017507 ENSCAFG00000018282 ENSCAFG00000018309 ENSCAFG00000019374 ENSCAFG00000019552 ENSCAFG00000019602 ENSCAFG00000019730 ENSCAFG00000019881 ENSCAFG00000023431 ENSCAFG00000025596 ENSCAFG00000030240 ENSCAFG00000031536 ENSCAFG00000032024 ENSCAFG00000032520 |
| System process | ENSCAFG00000001907 ENSCAFG00000003510 ENSCAFG00000009036 ENSCAFG00000010884 ENSCAFG00000011401 ENSCAFG00000016292 ENSCAFG00000016316 ENSCAFG00000019927 ENSCAFG00000023431 |
| Taxis | ENSCAFG00000018309 ENSCAFG00000019602 ENSCAFG00000025596 |
