# Supplementary material for: DNA methylation in canine brains is related to domestication and dog-breed formation
Source: PLoS One. 2020 Oct 29;15(10):e0240787. doi: 10.1371/journal.pone.0240787 (PMC7595415; doi:10.1371/journal.pone.0240787)
Supplement: S5 Table — Location is presented as chromosome:start-stop and adjacent regions have been merged. Strand is indicated by 1 or -1. Negative log fold change (FC) is less methylated in females and positive more methylated in females. (DOCX) [file pone.0240787.s005.docx]

**S5 Table. List of genes with differentially methylated regions between female and male dogs.** Location is presented as chromosome:start-stop and adjacent regions have been merged. Strand is indicated by 1 or -1. Negative log fold change (FC) is less methylated in females and positive more methylated in females.

| **Location** |  | **LogFC** | **Position** | **Gene id (Ensembl)** | **Symbol** | **Gene description** |
| --- | --- | --- | --- | --- | --- | --- |
| 2:82386601-82386900 | -1 | -4.3 | Intron | ENSCAFG00000016338 | KAZN | Kazrin, periplakin interacting protein |
| 3:60895601-60895700 | 1 | -4.1 | Upstream | ENSCAFG00000014627 | LOC102155289 | Elongation factor 1-alpha 1 |
| 3:60895601-60895700 | -1 | -4.1 | Intron | ENSCAFG00000014622 | DOK7 | Docking protein 7 |
| 5:4942601-4942700 | 1 | 3.5 | Upstream | ENSCAFG00000010199 | NFRKB | Nuclear factor related to kappa-B-binding protein |
| 5:4942601-4942700 | 1 | 3.5 | Downstream | ENSCAFG00000010164 | PRDM10 | PR/SET domain 10 |
| 5:82933101-82933400 | 1 | 4.7 | Intron | ENSCAFG00000035935 | - | *Uncharacterized* |
| 6:2558201-2558600 | 1 | 3.5 | Upstream | ENSCAFG00000030708 | - | *Uncharacterized* |
| 6:2558201-2558600 | -1 | 3.5 | Intron | ENSCAFG00000011150 | GALNT17 | Polypeptide N-acetylgalactosaminyl-transferase17 |
| 9:40807901-40808200 | -1 | 5.0 | Intron | ENSCAFG00000018462 | RNF135 | Ring finger protein 135 |
| 13:36963101-36963500 | 1 | 4.3 | Intron | ENSCAFG00000031517 | - | *Uncharacterized* |
| 16:55783001-55783500 | -1 | 4.2 | Intron | ENSCAFG00000008686 | CSMD1 | CUB and Sushi multiple domains 1 |
| 20:57562101-57562400 | 1 | 3.9 | Downstream | ENSCAFG00000023991 | CBARP | CACN subunit beta associated regulatory protein |
| 20:57562101-57562400 | -1 | 3.9 | Intron | ENSCAFG00000019538 | STK11 | Serine/threonine kinase 11 |
| 21:35980601-35981000 | 1 | 4.8 | Upstream | ENSCAFG00000008082 | RASSF10 | Ras association domain family member 10 |
| 24:47422601-47422900 | -1 | 3.5 | Intron | ENSCAFG00000013099 | UCKL1 | Uridine-cytidine kinase |
| 24:47422601-47422900 | -1 | 3.5 | Downstream | ENSCAFG00000013120 | ZNF512B | Zinc finger protein 512B |
| 28:40695301-40695600 | 1 | -3.9 | Downstream | ENSCAFG00000036910 | - | *Uncharacterized* |
| 28:40695301-40695600 | 1 | -3.9 | Intron | ENSCAFG00000031153 | - | *Uncharacterized* |
| 28:40695301-40695600 | 1 | -3.9 | Intron | ENSCAFG00000013430 | ZNF511 | Zinc finger protein 511 |
| 33:23285501-23285900 | -1 | 3.7 | Downstream | ENSCAFG00000030985 | POPDC2 | Popeye domain-containing protein 2 |
| X:122559301-122559500 | -1 | 4.0 | 3' UTR | ENSCAFG00000019613 | GAB3 | GRB2 associated binding protein 3 |
