# Supplementary material for: DNA methylation in canine brains is related to domestication and dog-breed formation
Source: PLoS One. 2020 Oct 29;15(10):e0240787. doi: 10.1371/journal.pone.0240787 (PMC7595415; doi:10.1371/journal.pone.0240787)
Supplement: S6 Table — (DOCX) [file pone.0240787.s006.docx]

| **S6 Table.** High level gene ontology categories from ShinyGO v0.50 for genes with DMRs from the comparison between female and male dogs. | |
| --- | --- |
| **High level GO category** | **Genes** |
| Biosynthetic process | ENSCAFG00000013099 ENSCAFG00000011150 ENSCAFG00000014627 ENSCAFG00000019538 |
| Immune response | ENSCAFG00000018462 ENSCAFG00000019538 |
| Immune system process | ENSCAFG00000018462 ENSCAFG00000019538 |
| Localization | ENSCAFG00000019538 ENSCAFG00000023991 |
| Negative regulation of biological process | ENSCAFG00000019538 ENSCAFG00000023991 |
| Positive regulation of biological process | ENSCAFG00000019538 ENSCAFG00000014622 ENSCAFG00000018462 |
| Regulation of biological quality | ENSCAFG00000008686 ENSCAFG00000019538 |
| Regulation of immune system process | ENSCAFG00000018462 ENSCAFG00000019538 |
| Regulation of localization | ENSCAFG00000019538 ENSCAFG00000023991 |
| Regulation of metabolic process | ENSCAFG00000019538 ENSCAFG00000014622 |
| Regulation of molecular function | ENSCAFG00000019538 ENSCAFG00000014622 ENSCAFG00000023991 |
| Regulation of multicellular organismal process | ENSCAFG00000018462 ENSCAFG00000019538 |
| Regulation of response to stimulus | ENSCAFG00000018462 ENSCAFG00000019538 |
| Response to stress | ENSCAFG00000018462 ENSCAFG00000019538 |
| Signaling | ENSCAFG00000019538 ENSCAFG00000008082 |
|  |  |
